# Curvature‐Directed Patch Formation on Gold Nanocubes by Thermally Induced Polymer Redistribution

**DOI:** 10.1002/advs.202510020

**Published:** 2025-09-08

**Authors:** Jaedeok Lee, Ignacio Pérez‐Juste, Jorge Pérez‐Juste, Isabel Pastoriza‐Santos, Semi Kim, Sang Yong Nam, Hyosung An, Luis M. Liz‐Marzán, Juyeong Kim

**Affiliations:** ^1^ Department of Chemistry Gyeongsang National University Jinju 52828 South Korea; ^2^ Research Institute of Advanced Chemistry Gyeongsang National University Jinju 52828 South Korea; ^3^ CINBIO Universidade de Vigo Campus Universitario Lagoas Vigo 36310 Spain; ^4^ Departamento de Química Física Universidade de Vigo Campus Universitario As Lagoas Vigo 36310 Spain; ^5^ Department of Materials Engineering and Convergence Technology Gyeongsang National University Jinju 52828 South Korea; ^6^ Department of Petrochemical Materials Engineering Chonnam National University Yeosu 59631 South Korea; ^7^ CIC biomaGUNE Basque Research and Technology Alliance (BRTA) Donostia‐San Sebastián Mendaro 20014 Spain; ^8^ Centro de Investigación Biomédica en Red, Bioingeniería Biomateriales y Nanomedicina (CIBER‐BBN) Donostia‐San Sebastián 20014 Spain; ^9^ Ikerbasque Basque Foundation for Science Bilbao 48009 Spain

**Keywords:** gold nanoparticle, morphology effect, patchy nanostructure, polymer ligand, shape‐anisotropy

## Abstract

Patchy nanoparticles (NPs) enable directional interactions and dynamic structural transformations, yet controlling polymeric patch formation with high spatial precision remains a significant challenge. Here, a thermally driven approach is presented to forming polystyrene (PS) patches on low‐curvature facets of anisotropic gold nanocubes (NCs) using a single polymer component. Heating in DMF above 90 °C triggers selective desorption of PS chains from high‐curvature edges and vertices via Au─S bond dissociation, followed by migration and deposition into rounded patches on flat surfaces. The number and angular configuration of patches are governed by NP geometry and thermal energy, typically appearing at 90° intervals. Patch morphology is highly responsive to NC concentration. Lower concentrations promote thicker convex patches, whereas higher concentrations suppress polymer rearrangement. Molecular dynamics simulations reproduce the curvature‐sensitive detachment and asymmetric redistribution observed experimentally. Solvent polarity and core composition serve as additional tools to modulate patch formation. This study reveals a robust mechanism for curvature‐directed polymer rearrangement and introduces a precise and scalable strategy for engineering patchy nanostructures. The ability to selectively pattern polymers onto specific nanoscale regions opens new opportunities for constructing programmable surfaces in plasmonics, catalysis, and anisotropic self‐assembly.

## Graphical abstract

Heating induces selective desorption and migration of polystyrene chains on anisotropic gold nanocubes, leading to the formation of discrete polymer patches exclusively on flat facets. Patch number and geometry are tunable by temperature and concentration. Molecular dynamics simulations support a curvature‐directed mechanism toward programmable nanostructures for plasmonics and self‐assembly.

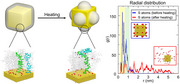

## 1 Introduction

Patchy nanoparticles (NPs) are an innovative type of multidimensional nanostructures, comprising core materials and adlayer constituents.^[^ ^1^, ^2^, ^3^, ^4^, ^5^ ^]^ They offer an expanded range of morphological diversity,^[^ ^6^, ^7^, ^8^, ^9^ ^]^ synergistic physicochemical properties among multiple components,^[^ ^10^, ^11^ ^]^ and geometric reconfigurability in response to external stimuli.^[^ ^12^, ^13^, ^14^ ^]^ For instance, the inclusion of patch components has been reported to induce directional patchy plasmonic NP assembly, leading to enhanced electric field, compared to their unpatched counterparts.^[^ ^15^, ^16^, ^17^ ^]^ Numerous synthetic approaches have been developed to integrate patches onto the core material, such as ligand‐mediated patch‐to‐core attachment,^[^ ^18^, ^19^ ^]^ direct growth of metallic patch NPs on high‐curvature areas (with high surface energy) of the core material,^[^ ^20^, ^21^ ^]^ or facet‐selective adsorption and transformation of grafting layers.^[^ ^22^ ^]^ Complementary bonding affinity with DNA as surface ligand could facilitate site‐selective attachment with patch NPs alongside core NPs,^[^ ^23^ ^]^ albeit these patchy structures may exhibit limited structural responsiveness. In addition, the direct formation of metallic patches is constrained to high‐curvature areas with high reactivity in shape‐anisotropic nanostructures.^[^ ^24^, ^25^ ^]^ The incorporation of grafting layers may enhance the material adaptability of the core NP to external stimuli, particularly in terms of its structural flexibility and the activation of highly reactive crystal facets.

Patchy NPs with grafting layers have conventionally been prepared using polymers as patch components on NP cores, usually accomplished through ligand‐selective surface adsorption, microphase separation, or manipulation of solvent polarity. As an example, the preferential adsorption of 2‐naphthalenethiols at the tips of gold nanoprisms allowed for the deposition of polystyrene‐*b*‐poly(acrylic acid) onto their high‐curvature regions, leading to a decrease of chemical activity at the nanoprism tips.^[^ ^26^, ^27^ ^]^ By applying different solvent environments, multiple polymeric patches have been obtained through microphase separation of binary mixed copolymer brushes grafted on silica NPs.^[^ ^28^ ^]^ In a similar vein, shape‐anisotropic gold nanopolyhedra grafted with thiol‐terminated polystyrene (PS) were shown to undergo distinct polymer shell segregation, particularly at their sharp areas, upon an increase in solvent polarity.^[^ ^29^, ^30^, ^31^ ^]^ While these approaches result in the formation of polymer patches, they present limitations such as obscuring high‐curvature facets important for surface reactivity, restricting access to catalytically active sites, and relying on expensive block copolymers, which limit their scalability and practical application. Strategies enabling patch formation on low‐curvature surfaces may help address these issues, but mechanistic insights into such processes remain limited. Although few studies have reported patch formation on low‐curvature regions,^[^ ^12^, ^32^ ^]^ these instances remain exceptionally rare and lack mechanistic clarity. Furthermore, to the best of our knowledge, surface‐selective patch formation using a single polymer component has not been demonstrated, underscoring a critical gap in selectively targeting high‐curvature regions.

Here, we present a single‐step method for the formation of polymer patches selectively on low‐curvature facets, starting from PS‐grafted gold nanocubes (PS‐NCs). In contrast to previous studies where PS‐NCs predominantly displayed polymer shells at tips and edges in response to changes in solvent polarity,^[^ ^29^, ^30^, ^31^ ^]^ our approach favors shells on flat surfaces and thus exposure of high‐curvature surfaces through thermal activation. Upon heating above 90 °C, Au─S bond dissociation occurs preferentially at high‐curvature edges and tips, leading to the desorption of PS chains, which subsequently migrate and reattach onto low‐curvature facets, forming rounded patches. This curvature‐dependent dissociation originates from the differential polymer structures imposed by surface curvature. At high‐curvature regions, the PS chains with mushroom‐like structure exhibit features such as reduced interchain interactions and higher conformational entropy, which contribute to the weakening of Au─S bond stability. This spatial redistribution is governed by NP geometry and thermal energy, resulting in patch coordination numbers from one to six, with interpatch angles toward multiples of 90°. Molecular dynamics (MD) simulations support this mechanism by demonstrating curvature‐dependent desorption and redistribution of PS chains. Whereas previous studies reported heat‐induced reconfiguration of polymer shells on gold NPs, these systems typically involved post‐formation restructuring of patches that were initially generated through solvent‐driven phase separation^[^ ^33^, ^34^ ^]^ or relied on passive shell contraction.^[^ ^31^, ^32^ ^]^ Our approach enables active and directional redistribution of surface polymers through thermal activation. To confirm this curvature‐dependent trend, we further applied our method to gold concave nanocubes (CCs) with higher local curvature and consistently observed localization of patches on low‐curvature surfaces.^[^ ^35^ ^]^ Additionally, we found that the number and morphology of patches, as well as the lifted orientation of the NCs on substrates, can be tuned by varying parameters such as temperature, reaction time, and NP concentration. This highlights the thermally programmable control over patch geometry and nanoscale orientation. Together, these findings demonstrate a robust and versatile approach for selective patch formation via thermally mediated polymer migration, uniquely targeting low‐curvature regions of anisotropic NPs.

## 2 Results and Discussion

**Figure 1:**
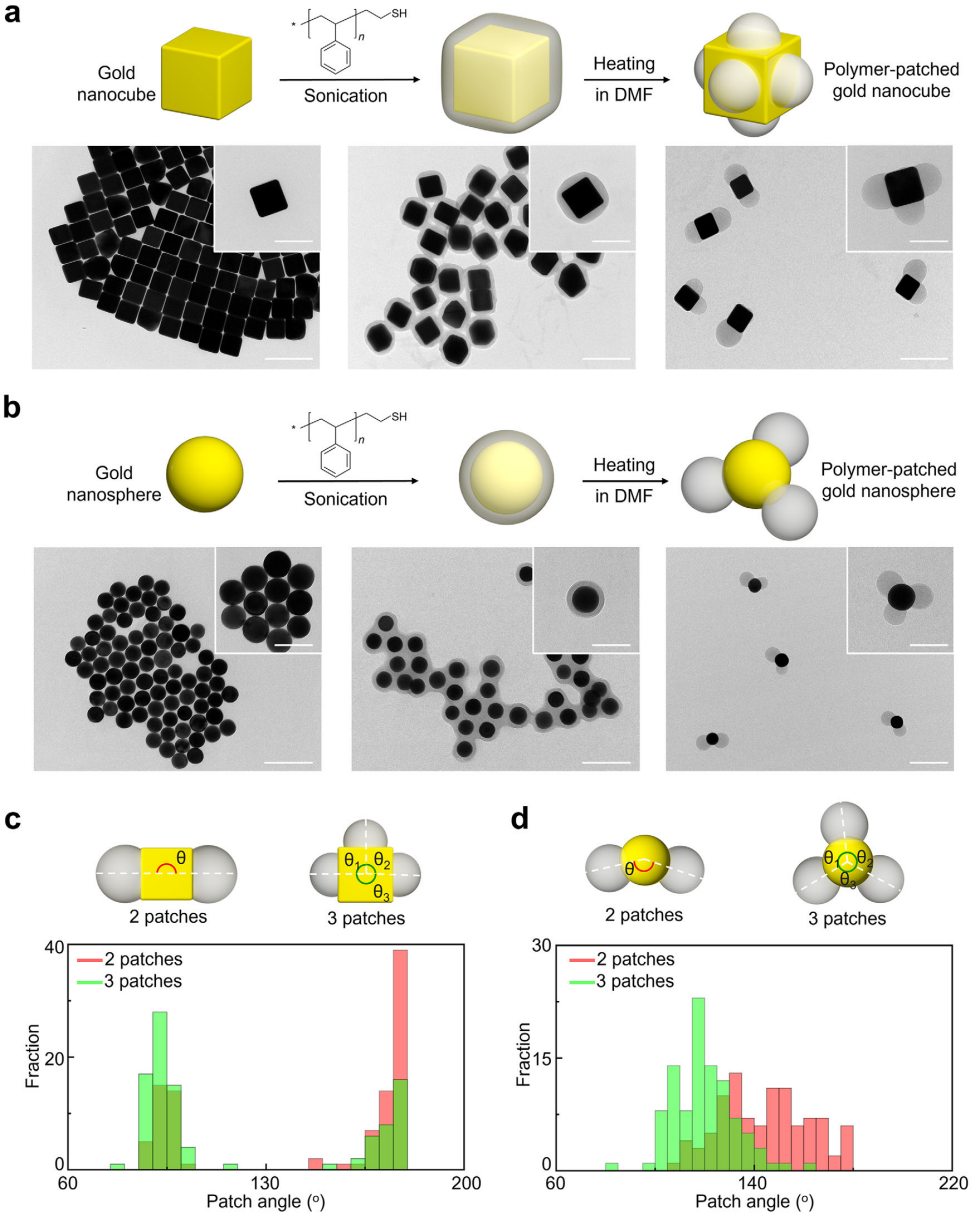
a) Schematic of PS grafting and patch formation of gold NCs and TEM images of gold NCs (left), PS‐NCs (middle), and patchy PS‐NCs (right). b) Schematic of PS grafting and patch formation of gold NSs and TEM images of gold NSs (left), PS‐NSs (middle), and patchy PS‐NSs (right). c) Schematic of PS‐NC with two and three patches, where the patchy angles (θ_x_) are illustrated (top). Patch angle distribution histogram measured from patchy PS‐NCs with two (red) and three (green) patches (bottom). d) Schematic of PS‐NS with two and three patches where the patchy angles (θ_x_) are illustrated (top). Patch angle distribution histogram measured from patchy PS‐NSs with two (red) and three (green) patches (bottom). Scale bars: 200 nm for (a) and (b), and 100 nm for (a) and (b) insets.

Gold NCs (86.3 ± 3.4 nm in edge length) with high monodispersity in shape and size were prepared via a seed‐mediated growth method (see details in Figure S1, Supporting Information).^[^ ^36^ ^]^ They were chosen for their well‐defined shape, featuring six flat surfaces, twelve sharp edges, and eight sharp corners. The edges and corners provide high surface reactivity, making them ideal for catalysis and sensing applications. The symmetry of the NC structure also allows for consistent comparisons of surface modifications, which is essential for examining the effects of polymer grafting on the functional properties of nanostructures. Prior to polymer adsorption, cetyltrimethylammonium bromide (CTAB) surfactant molecules as the gold NC capping agent were removed from the gold NC dispersion through iterative centrifugations, achieving the CTAB concentration of less than 500 µm. The PS grafting carried out via quick injection of the NCs into a thiol‐terminated PS (*M_n_* = 50000 g mol^−1^) solution in *N*,*N*‐dimethylformamide (DMF) under sonication.^[^ ^37^ ^]^ Transmission electron microscopy (TEM) images of PS‐NCs revealed a core–shell structure comprising the gold core appearing in dark contrast and the PS shell in lighter contrast (**Figure** **1**a; Figure S1, Supporting Information). This indicates that the PS shell uniformly covered the surface of the gold NC, with an average shell thickness of 19.6 ± 1.9 nm. The extinction spectra of gold NCs before and after PS grafting showed a red‐shift of the localized surface plasmon resonance from 595 to 625 nm (Figure S1b; Figure S1e, Supporting Information), confirming the PS grafting on the surface of gold NCs.

The formation of polymer patches on PS‐NCs was induced by simply heating the PS‐NCs dispersion in DMF above 90 °C (see details in Supporting Information). The TEM analysis showed that the uniform PS layers on PS‐NCs transformed into segregated patchy layers located on the NC planar facets (Figure 1a). For comparison, polymer grafting and patch formation were also applied to gold nanospheres (NSs) of similar size (Figure 1b). In all patchy PS‐NCs, polymer patches were observed to form at periodic angles in multiples of 90°, suggesting that the emergence of such patches is governed by the cubic core geometry (Figure 1c; Figure S2a, Supporting Information). The analysis of the patch angle distribution revealed that patchy PS‐NCs with two patches exhibited angles of 90° determined in 35% of cases and 180° in 65% of cases. The prevalence of 180° angle between patches suggests that repulsion forces between them drive the formation of this configuration.^[^ ^38^, ^39^ ^]^ For PS‐NCs with three patches, the angle distribution showed a higher proportion of 90° angles (67%) compared to 180° angles (33%), due to the additional patch occupying a planar facet of PS‐NCs. As a comparison, the patch angles on the isotropic (spherical) gold cores were found to be determined by the number of patches (Figure 1d; Figure S2b, Supporting Information), rather than the shape of the core. The average patch angle on the patchy PS‐NSs with two patches was 144 ± 17.9°, while in particles with three patches it was 120 ± 12.6°. As a result, the physically separated domains on NCs due to their curvature reduced repulsion of patches, leading to interpatch angles that were independent of number of patches. In contrast, NSs with milder curvature allowed interaction of patches, resulting in a decrease in interpatch angle with increasing number of patches. The observation that polymer patches form on low‐curvature facets of gold NCs suggests that these regions are energetically favorable.^[^ ^15^ ^]^ Intervening high‐curvature domains physically separate such facets and reduce interpatch repulsion. By contrast, AuNSs lack distinct separating domains, allowing patches to form across the entire surface. The resulting steric repulsion may influence their spatial arrangement, which is consistent with the variation in interpatch angle with patch number (Figure 1d; Figure S2b, Supporting Information). These initial results suggest that the anisotropy of the metal core plays a significant role in modulating the adjacent angle between polymer patches.

To further examine the influence of curvature on patch localization, we extended our study to CCs, which expose {720} facets with higher local curvature than the flat {100} facets of conventional NCs (Figure S3, Supporting Information).^[^ ^35^ ^]^ TEM analysis revealed that polymer patches preferentially formed on the relatively low‐curvature facets of the CCs, whereas high‐curvature tips and concave regions did not exhibit any polymer shell. This result further supports the curvature‐dependent desorption and reattachment mechanism of PS chains proposed in this study. We also investigated the effect of polymer chain length as another factor influencing patch formation (Figure S4, Supporting Information). Experiments with thiol‐terminated PS of lower molecular weights (*Mn* = 11 500 and 25 000 g mol^−1^) revealed a clear dependence on chain length. For PS with *Mn* = 11 500 g mol^−1^, heating at 90 °C for 2 h resulted in desorption of the polymer from the NP surface without detectable patch formation. In contrast, PS with *Mn* = 25 000 g mol^−1^ produced distinct patches, albeit smaller in size and predominantly formed on low‐curvature surface regions. These findings indicate that 25 000 g mol^−1^ PS chains provide sufficient chain entanglement and cohesive interactions to enable partial patch formation, whereas 11 500 g mol^−1^ chains do not reach the threshold length required for such formation.

**Figure 2:**
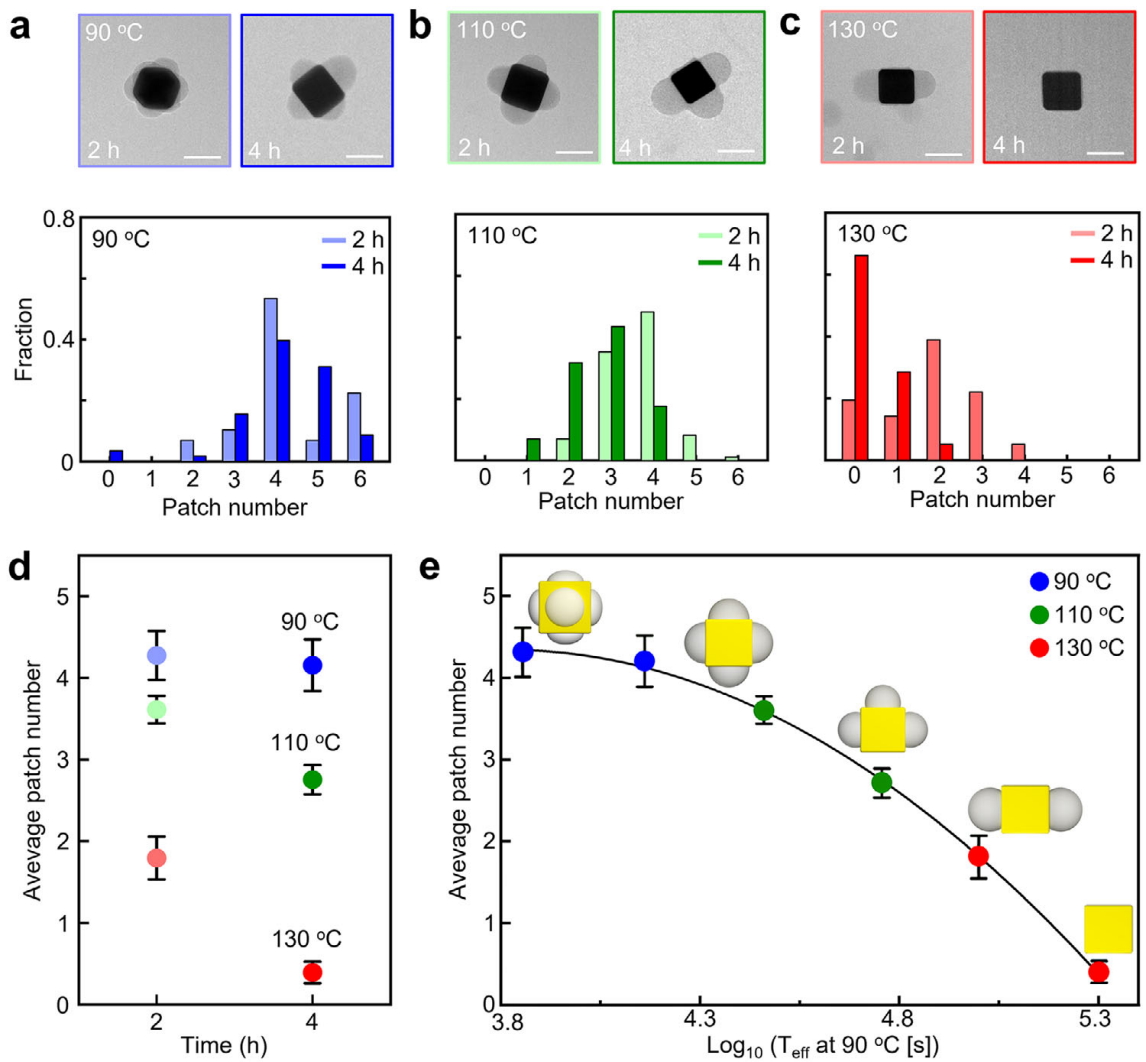
Representative TEM images of patchy PS‐NCs obtained after heating treatment at 90 °C a), 110 °C b), and 130 °C c), and their corresponding patch number distribution graphs. At each temperature, patch formation proceeded for either 2 h (light‐colored) or 4 h (dark‐colored). d) A plot of average patch number per particle (color code is the same as in a–c) and e) master curve obtained from TTSP of different heating temperatures and times. The illustrations show representative patchy NPs for each patch number. Scale bars: 100 nm.

Interestingly, the number of polymer patches within PS‐NCs could be systematically adjusted by varying the heating treatment (temperature and time) (**Figure** **2**). The heating temperature was varied from 90 to 130 °C in increments of 20 °C, with heating times ranging from 2 to 4 h. After heat treatment, the PS‐NC colloid was left at room temperature for 1 h before TEM characterization. The number of patches on the surface of the gold core was counted from TEM images for each condition and compared in distribution plots of the number of patches. At 90 °C, the number of patches ranged between 4 and 6 (Figure 2a; Figure S5, Supporting Information). Over a heating treatment of 2 to 4 h, the population with 6 patches decreased by 62%, while the patchy PS‐NCs with 5 patches increased 4.5‐fold. Overall, the average number of patches slightly decreased from 4.3 by 2 h to 4.2 by 4 h at 90 °C (Figure 2d). This effect was even more pronounced at higher temperatures. For example, after heating at 110 °C, the population of patches shifted to even lower numbers (Figure 2b; Figure S6, Supporting Information), with a 63% decrease in NCs with 4 patches along with a 5.5‐fold increase in the NCs with 2 patches, from 2 to 4 h. The average number of patches also decreased significantly, from 3.6 at 2 h to 2.7 at 4 h (Figure 2d). At an even higher temperature of 130 °C, the dominant distribution of patch numbers ranged between 0 and 3 (Figure 2c; Figure S7, Supporting Information). After patch removal, a small amount of residual polymer layer remained on the surface. The average number of patches at 130 °C was the smallest as compared to those at the other temperatures (Figure 2d), which we attribute to excessive thermal energy leading to detachment of patches from the NPs surface. To quantitatively interpret the effect of both temperature and time on patch evolution, the time‐temperature superposition principle (TTSP) based on the Arrhenius equation was applied to the data in Figure 2d.^[^ ^40^ ^]^ This approach enabled the integration of time‐dependent data across different thermal conditions into a single master curve, thereby revealing that the patch formation process is governed by a unified thermally activated mechanism (Figure 2e). The successful superposition of all datasets confirms that the temperature and time‐induced decrease in patch number follows a common kinetic pathway characterized by a consistent activation energy. By applying TTSP to rescale all experimental data onto a reduced time axis, we not only clarified the thermal dependence of the patch reorganization process but also enhanced the mechanistic understanding of polymer migration dynamics on anisotropic NP surfaces.

**Figure 3:**
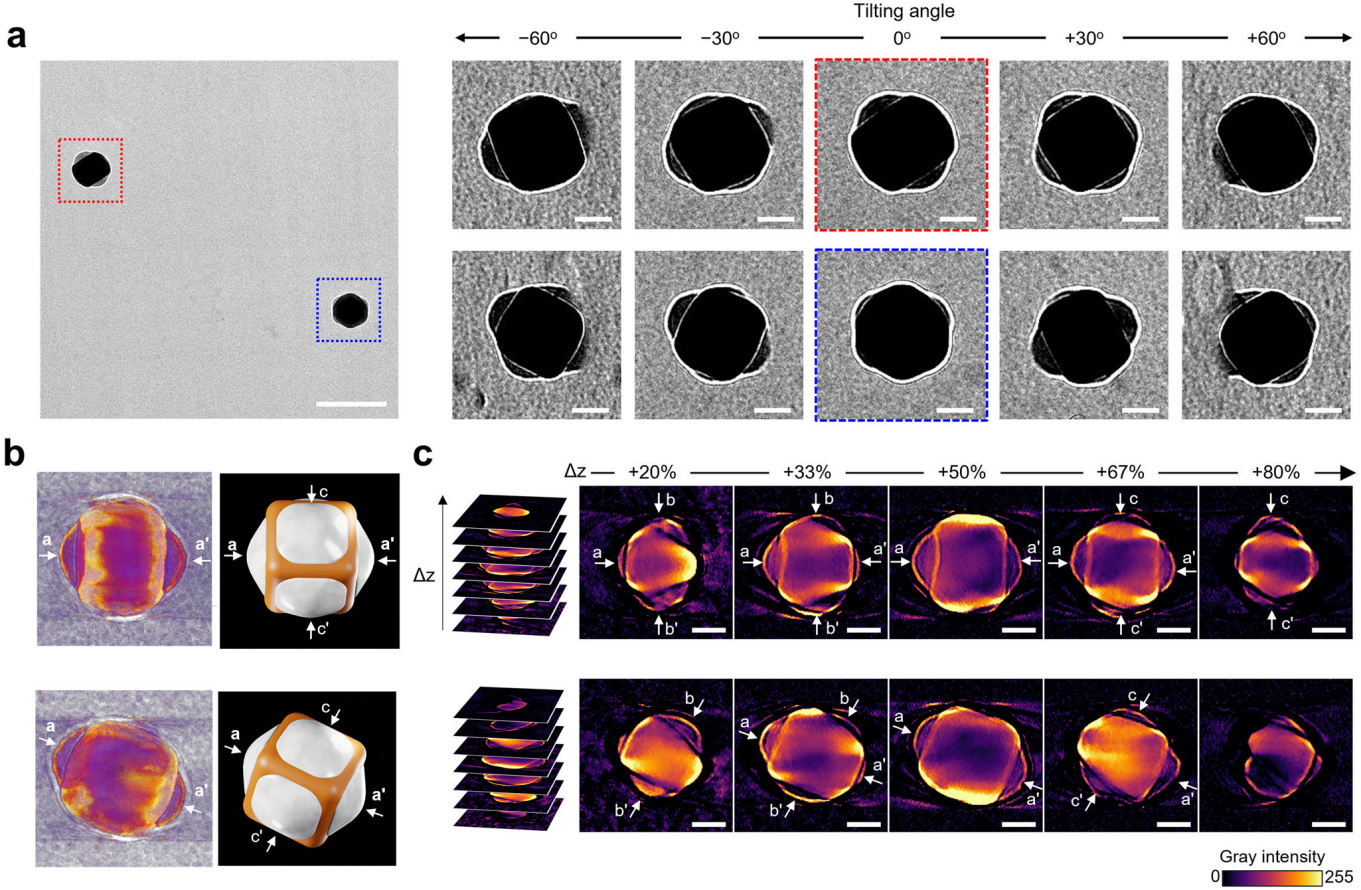
a) A representative TEM image of patchy PS‐NCs obtained after heating at 90 °C for 2 h, along with TEM tilt series (5 out of 61 total) acquired at various tilt angles from −60° to +60°. b) 3D reconstruction images and corresponding schematic models. c) The z‐slices of the reconstructed tomograms of (b). Scale bars: 200 nm for (a, left), 50 nm for (a, right), and (c).

It should be noted that, since the number of patches was counted using 2D TEM images, some patches might be hidden by the NC core, resulting in slight under‐counting of the total PS hemisphere count per particle. This effect, however, is expected to occur randomly and thus not significantly alter the observed statistical trends (Figure S8, Supporting Information). We therefore performed TEM tomography to get inside the 3D location and morphology of the patches (**Figure** **3**). A series of TEM images was obtained at different angles ranging from −60° to +60° for the patchy PS‐NCs formed at 90 °C for 2 h (Figure 3a; Movie S1, Supporting Information). The 3D structure of the patchy PS‐NCs was reconstructed from 61 projections, indicating the formation of rounded polymer patches on six planar facets (Figure 3b; Movie S2, Supporting Information). These results were complemented by visualized 2D imaging (z‐slice) obtained from 3D modeling of the tomographic data (Figure 3c; Figure S9, and Movie S3, Supporting Information). In the upper z‐slice series of Figure 3c, at Δz = +20%, three patches—designated as a, b, and b'—are observed at the bottom of the NC core. At Δz = +33%, an additional patch, a', appears. Subsequently, the b and b' patches disappear at Δz = +50%, while new patches, c and c', emerge at Δz = +67%. These observations indicated that a total of six patches are formed along the z‐axis of the NPs core.

Based on these findings, we propose a model to elucidate the temperature‐ and time‐dependent patch formation in PS‐NCs (Figure S10, Supporting Information). At the moderate temperature of 90 °C, thermal energy is hypothesized to disrupt the bonds between gold atoms on the NC surface and thiols within the PS layer, thereby facilitating polymer chain displacement. According to literature, polymer grafted on gold NPs can form either brush‐type or mushroom‐type structures, depending on the grafting density and substrate curvature.^[^ ^41^ ^]^ The mushroom configuration is characterized by higher steric freedom and better interaction with the solvent, compared to the brush configuration, which leads to lower glass transition temperature and greater detachment from the NP surface.^[^ ^42^ ^]^ We assume that PS layers on PS‐NCs are in a brush‐type structure on flat facet surfaces, but form a mushroom‐type structure on tips and edges (Figure S11, Supporting Information). Upon heating, initial partial desorption of thiolated PS molecules is anticipated to occur predominantly from high‐curvature regions, such as tips and edges, due to increased steric strain and weaker adhesion forces. The desorbed PS chains are expected to associate with existing polymer domains in low‐curvature regions through chain entanglement and *π*–*π* interactions, rather than re‐adsorbing onto the gold surface to form new Au─S bonds. Simultaneously, the brush‐type PS layer adjacent to the desorption area may undergo a structural transition from a brush to a mushroom configuration. This transition is driven by the partial removal of neighboring polymer chains, which reduces the local grafting density, increases steric freedom, and enhances solvent‐polymer interaction. Over time, the pre‐formed patches experience additional deformation caused by thermally induced rearrangement of polymer chains, ultimately culminating in the formation of rounded patches on the planar facets of the gold NC. At elevated temperatures of 110 and 130 °C, the initial patch desorption from the gold NC surface occurs before the released polymer chains can fully rearrange and re‐adsorb, indicating a temperature threshold beyond which thermal energy overcomes polymer adhesion. This patch formation mechanism was supported by the observation of PS desorption and reattachment when mechanical stirring was introduced (Figure S12, Supporting Information). More specifically, PS‐NCs were heated at 90 °C for 2 h under mechanical stirring at 100 rpm. Stirring appeared to hinder re‐adsorption of released PS, leading to its complete detachment. In contrast, the same heating condition without stirring promoted the formation of multiple rounded patches on the gold NC.

**Figure 4:**
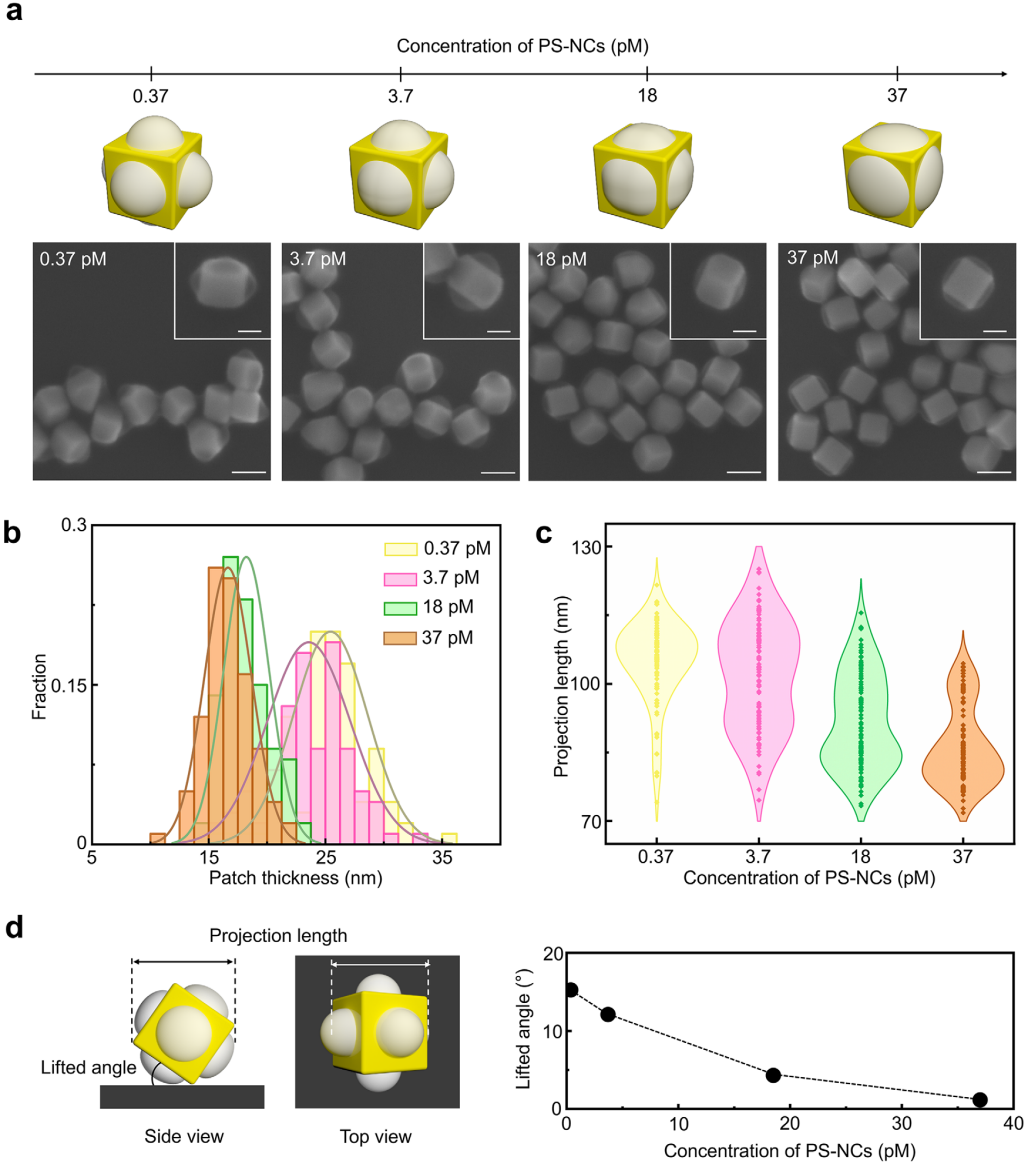
a) Schematic illustrations and representative SEM images of the patchy PS‐NCs obtained after heating colloids with different concentrations of PS‐NCs at 90 °C for 2 h. b) Patch thickness distribution histogram of the patchy PS‐NCs formed at different concentrations of PS‐NCs. c) Violin plot of the projection length distribution of the patchy PS‐NCs formed at different concentrations of PS‐NCs. d) Schematic illustrations showing the side and top views of a patchy PS‐NC on the flat substrate, and a plot of lifted angle as a function of PS‐NC concentration. Scale bars: 100 nm for (a) and 50 nm for (a) inset.

We also found that the thickness and morphology of polymer patches within PS‐NCs depended on the concentration of PS‐NCs (**Figure** **4**). Colloidal dispersions with PS‐NC concentrations ranging from 0.37 to 37 pm (in terms of gold NCs) were heated at 90 °C for 2 h. Note that these experiments were performed in the absence of free polymer, using only PS‐NCs dispersed in DMF, thereby maintaining a constant polymer‐to‐NP ratio across all concentrations. Scanning electron microscopy (SEM) characterization of the resulting patchy PS‐NCs indicated that the morphology of patches changed from rounded to increasingly flattened as the concentration of PS‐NCs increased (Figure 4a), where the brush‐type structure was well maintained while the mushroom‐type PS layer was desorbed minimally for 37 pm. This hindered an increase in patch thickness with concentration: 25.4 ± 3.2 nm for 0.37 pm, 23.6 ± 3.6 for 3.7 pm, 18.2 ± 1.9 for 18 pm, and 16.7 ± 2.0 nm for 37 pm (Figure 4b; Figure S13, Supporting Information). At the highest concentration, the patchy PS‐NCs appeared to undergo only partial desorption of the PS layer in confined spaces with high curvature.

To determine whether the sampling process could affect polymer patch formation and morphology, we performed two complementary analyses. First, we investigated whether trace amounts of water during the sampling process could affect patch formation. As previously reported, trace amounts of water can induce a transition from core–shell to patchy morphologies.^[^ ^29^ ^]^ To rule out this possibility, the samples were rapidly dried under vacuum immediately after collection. Patches were consistently observed on low‐curvature surfaces regardless of the drying method, indicating that the sampling and drying procedures did not influence patch formation (Figure S14, Supporting Information). Also, to verify whether the high vacuum conditions during SEM analysis could affect the polymer patch morphology, we performed environmental‐SEM analysis (Figure S15, Supporting Information). The patchy PS‐NCs were imaged under low‐vacuum (10^3^ Pa), exposed to high‐vacuum (10^−3^ Pa) for ≈30 min, and then re‐imaged under low‐vacuum. No significant morphological changes were observed in the polymer patches.

Next, the orientation of the patchy PS‐NCs on the substrate was examined by SEM. As shown in the illustration from Figure 4d, we defined a lifted angle, related to the convexity of the patches. The projection length of the gold NC was measured along its long axis to extract the lifted angle (Figure 4c; Figure S16, Supporting Information). As the concentration of PS‐NCs increased, the projection length of the gold NC in SEM decreased (104.9 ± 8.9 nm for 0.37 pm, 101.8 ± 10.3 nm for 3.7 pm, 91.5 ± 12.0 nm for 18 pm, and 86.2 ± 8.9 nm for 37 pm) and consequently the lifted angle gradually decreased (Figure 4d). These findings indicate that the lifted orientation of patchy PS‐NCs on the substrate evolves in correlation with the development of the patches.

The concentration effect of PS‐NCs on patch thickness and NC orientation qualitatively supports the above proposed mechanism. Although direct quantitative measurement of PS release per particle was beyond the scope of the current study, we presumed that heating the PS‐NC dispersion at 90 °C facilitates the release of PS from high‐curvature areas. At higher concentrations of PS‐NCs, smaller amounts of PS are released per particle, quickly reaching an equilibrium PS concentration in solution. This equilibrium restricts the amount of PS released per particle and suppresses patch formation. Conversely, lower concentrations of PS‐NCs allow for the release of larger amounts of PS per particle and facilitate their reattachment through hydrophobic attraction between polymer chains, resulting in the formation of rounded patches with high convexity. This interpretation is further supported by image‐based quantification of patch contact area (Figure S17 and Table S1, Supporting Information). The average contact diameter increased from 60.6 nm at 0.37 pm to 71.1 nm at 37 pm, corresponding to a surface coverage increase from 38.7% to 53.3%. These results support the idea that reduced particle concentration facilitates polymer redistribution, enhancing patch formation on planar facets.

Upon the thermally‐driven release of PS from PS‐NCs, the solubility of the PS can affect the patch formation. To gain insight, experiments adding toluene as co‐solvent to the PS‐NC dispersion in DMF (1:1 in volume ratio) were conducted. Upon heating the PS‐NC dispersion at 90 °C for 2 h, fewer numbers of rounded patches per particle were formed and a significant quantity of free PS chains was observed on the TEM grid (Figure S18, Supporting Information). The superior solubility of PS in toluene, as compared to DMF,^[^ ^43^ ^]^ may inhibit the reattachment of released PS chains onto the surfaces of PS‐NCs during the heating process. Thus, solvent affinity is crucial to control the mobility of polymer chains and regulate patch formation.

In addition, the dissociation of the bond between thiolated‐PS and gold atoms at the PS‐NCs can be contrasted by using a different core component, such as silver. Desorption of thiol ligands from a silver surface is known to be more difficult than from a gold surface due to the higher polarity of the Ag─S bond and the strong van der Waals interactions between the surface ligands.^[^ ^44^, ^45^ ^]^ We conducted heating the solution of PS‐grafted silver‐coated gold NCs (PS‐Au@Ag NCs) at 90 °C for 2 h. The TEM images of PS‐Au@Ag NCs showed no patch formation (Figure S19, Supporting Information), with only trace amounts of spherical polymer micelles observed, whereas PS‐NCs displayed distinct patch formation at the same temperature. This finding also supports our proposed mechanism for patch formation, which involves the desorption and reattachment of PS.

**Figure 5:**
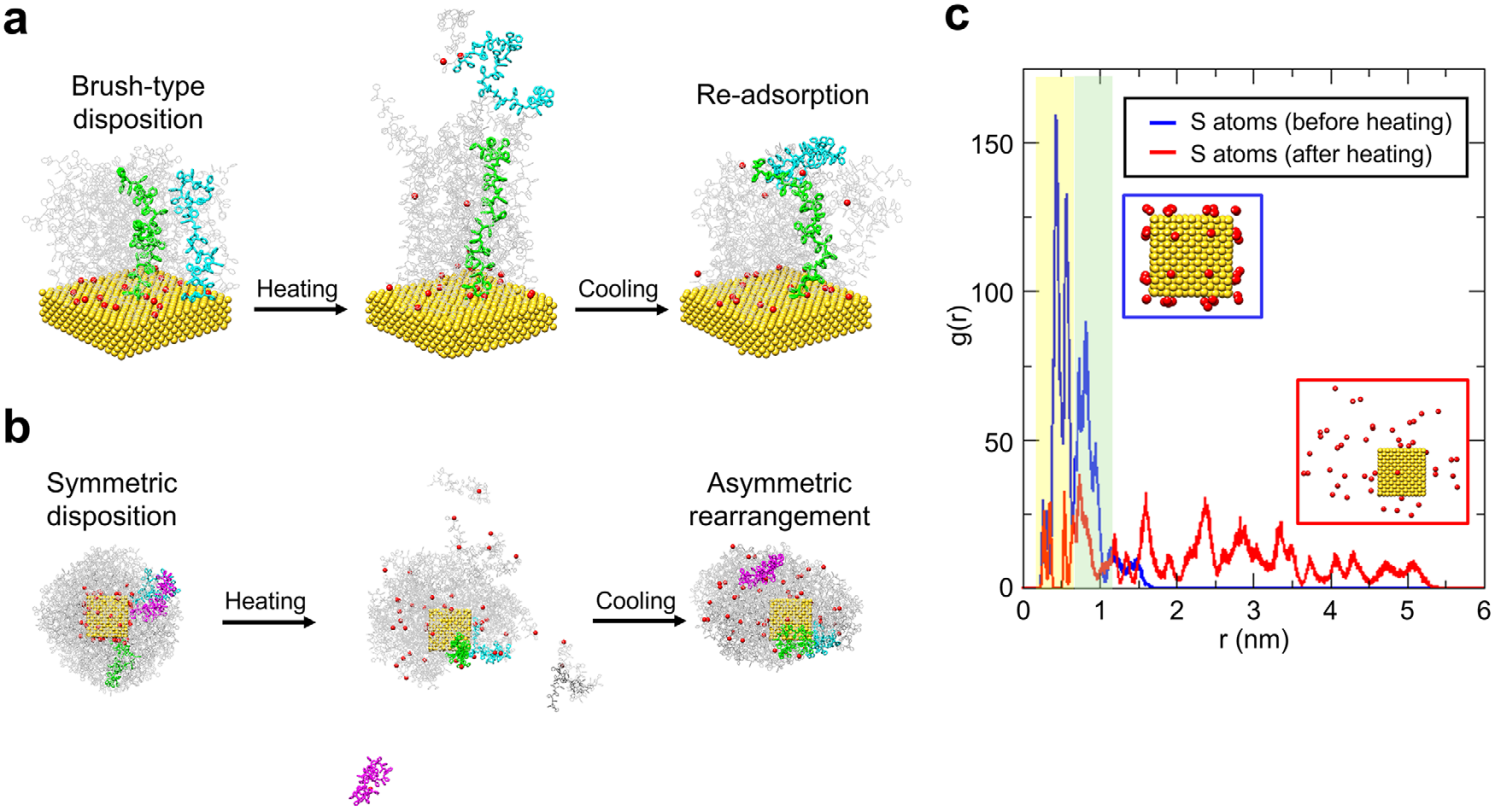
a) Selected snapshots for MD simulations of 20 PS chains adsorbed on gold: (left) initial equilibrated disposition at room temperature for PS chains adopting a brush‐type disposition, (middle) intermediate arrangement during the heating cycle, and (right) final equilibrated configuration with PS chains not directly attached to the surface interacting with another polymer chains. PS chains are represented in gray color, except those highlighted in cyan, green, and magenta that show the disposition of particular PS chains at different stages of the simulation. Sulfur atoms are depicted as red spheres. b) Selected snapshot for MD simulations of 48 PS chains adsorbed on a gold NC: (left) initial symmetric disposition at room temperature for PS chains adopting a brush‐type disposition, (middle) intermediate arrangement at the end of the heating step showing the detaching of the polymer, and (right) final asymmetric configuration around the metal NP after cooling down to the initial temperature. c) Equilibrium radial distributions of the sulfur atoms around the center of the gold NC at room temperature before (blue line) and after (red) heating the system. Distribution maxima in the yellow region correspond to positions of the sulfur atoms bonded on the faces of the cube and maxima in the green region correspond to sulfur bonded on the edges of the cube.

To get additional insight into the behavior of PS absorbed on gold, we performed MD simulations using two simple brush models. The first model involved 20 PS chains (40 monomeric units) interacting with an Au(100) slab of 5.3 × 5.3 × 1.1 nm^3^ containing 2028 gold atoms (**Figure** **5**a) and the other 48 PS chains interacting with a gold NC with an edge length of 2.5 nm (Figure 5b). To mimic our experimental procedure, after initial equilibration at room temperature, both models were heated until the PS chains detached from the metal surface and then cooled down to the initial temperature (see details for the MD simulations in Section S10 and Table S2, Supporting Information).

The MD simulations showed for PS grafted on a Au(100) slab (which can be considered the limit of high‐curvature for the metal surface) that PS chains adsorbed in a brush‐like arrangement adopting more stretched conformations than in the free polymer (Figure 5a). The average polymer end‐to‐end distance (*R*_ee_) was ≈50% larger in the 20 PS adsorbed on the gold surface compared to the free polymer (4.73 vs 3.15 nm in Table S3, Supporting Information). The stretched conformations of the adsorbed PS chains were also consistent with the anisotropy of the components of the radius of gyration (*R*_g_) in Table S3 (Supporting Information). After heating, MD simulations predicted that ≈20% of the PS chains detached from the gold surface and, after the final cooling step, these chains did not re‐adsorb onto the surface but remained interacting with the adsorbed polymers, adopting much more coiled conformations. As a consequence, the average *R*_ee_ in this final disposition was only ≈25% longer than in the free polymer (4.03 vs 3.15 nm in Table S3, Supporting Information) and there was also a significant reduction of the asymmetry of the *R*_g_ components. Therefore, MD simulations suggested that, upon heating and cooling, the polymer may detach from the metallic NP and be redistributed in the vicinity of the surface by maintaining interactions with neighboring chains, despite not being directly adsorbed on the surface.

For the 48 PS chains interacting with a gold cube, MD simulations after initial equilibration at room temperature indicated that the polymer distributed symmetrically around the metal NP (Figure 5b). Interestingly, although the grafting density for this model was slightly higher than for the flat one, both the *R*_ee_ and *R*_g_ values indicated that the PS chains adsorbed on the metal NP were somewhat shorter than those corresponding to the free polymer of similar size (*R*_ee_ values are 2.69 and 3.58 nm, respectively, in Table S3, Supporting Information). This trend reflects the fact that the area and volume available for the PS chains grafted to the NC are larger than in the flat model. In particular, edge regions with higher curvature may promote conformational constraints or preferential desorption, consistent with our experimental observations.

After heating, MD results indicated that some of the PS chains detached from the cube surface and, after the final cooling step, these chains remained around the NP through interactions with their counterparts. Remarkably, this new distribution around the NP is asymmetric (Figure 5c), similarly to what is observed experimentally. Due to the larger entanglement of the polymer and the larger volume available, the average conformational size of the PS chains is almost equal to that corresponding to the free polymer, as suggested by the similar values obtained for *R_g_* and its components (1.36 vs 1.41 nm in Table S3, Supporting Information). MD simulations also showed that, after the polymer release by heating, the lateral branches of some of the PS chains interacted directly with the remaining available space on the surface (Figure S20, Supporting Information), preventing the re‐adsorption of the detached molecules. MD simulations performed for metal NPs with different sizes and shapes confirmed the release/reattach pattern giving rise to final asymmetrical PS distributions (Figure S21, Supporting Information). To summarize, MD simulations suggest that after completion of a heating‐cooling cycle, a fraction of the PS chains adsorbed on the gold surface may detach from the surface and redistribute asymmetrically around the metal surface by means of intermolecular interactions with their neighboring polymer chains.

## 3 Conclusion

In this study, we demonstrated the controlled formation of patchy nanostructures using thermal energy applied to PS‐NCs. We found that polymeric patches formed exclusively on the low‐curvature surfaces of gold NCs, leaving high‐curvature areas exposed. Various parameters, including heating temperature, duration, and particle concentration, were investigated to control the number and characteristics of patches and to elucidate the underlying mechanism of patch formation. Increasing the heating temperature and duration led to a reduction in the number of patches, with counts decreasing from six to zero. Additionally, we modulated the thickness of the patches by varying the concentration of PS‐NCs; higher concentrations resulted in thinner patches due to the rapid equilibrium of released PS in solution, thereby reducing the release of PS per particle. The composition of the solvent also played a crucial role in patch formation. Specifically, a mixture of toluene with DMF failed to produce patches under identical conditions, highlighting the importance of the reattachment process of the PS released for patch formation. Confirmatory experiments with PS‐Au@Ag NCs supported our hypothesis that patch formation is initiated by the desorption of polymer chains, facilitated by thermal energy sufficient to break Au─S bonds and accelerate polymer migration. MD simulations further supported the proposed mechanism by demonstrating that polymer chains preferentially detached from high‐curvature regions upon heating and redistributed asymmetrically around the core. This behavior is consistent with the localized patch formation observed experimentally. Our research delivers pivotal insights that are essential for directing the synthesis and subsequent morphological progression of patchy NPs.

## Conflict of Interest

The authors declare no conflict of interest.

## Supporting information



Supporting Information

Supplemental Movie 1

Supplemental Movie 2

Supplemental Movie 3

## Data Availability

The data that support the findings of this study are available from the corresponding author upon reasonable request.
