# Supplementary material for: Curvature‐Directed Patch Formation on Gold Nanocubes by Thermally Induced Polymer Redistribution
Source: Adv Sci (Weinh). 2025 Sep 8;12(44):e10020. doi: 10.1002/advs.202510020 (PMC12667489; doi:10.1002/advs.202510020)
Supplement: Supplementary file 1 — Supporting Information [file ADVS-12-e10020-s003.pdf]

Supporting Information

Curvature-Directed Patch Formation on Gold Nanocubes by Thermally Induced Polymer Redistribution

Jaedeok Lee[a,b], Ignacio Pérez-Juste[c,d], Jorge Pérez-Juste[c,d], Isabel Pastoriza-Santos[c,d], Semi Kim[a,b], Sang Yong Nam[e], Hyosung An*[f], Luis M. Liz-Marzán*[c,g,h,i] and Juyeong Kim*[a,b]

[a]Department of Chemistry, Gyeongsang National University, Jinju 52828, South Korea

[b]Research Institute of Advanced Chemistry, Gyeongsang National University, Jinju 52828, South Korea

[c]CINBIO, Universidade de Vigo, Campus Universitario Lagoas, Vigo 36310, Spain

[d]Departamento de Química Física, Universidade de Vigo, Campus Universitario As Lagoas, Vigo, 36310, Spain

[e]Department of Materials Engineering and Convergence Technology, Gyeongsang National University, Jinju 52828, South Korea

[f]Department of Petrochemical Materials Engineering, Chonnam National University, Yeosu 59631, South Korea

[g]CIC biomaGUNE, Basque Research and Technology Alliance (BRTA), Donostia-San Sebastián 20014, Spain

[h]Centro de Investigación Biomédica en Red, Bioingeniería, Biomateriales y Nanomedicina (CIBER-BBN), Donostia-San Sebastián 20014, Spain

[i]Ikerbasque, Basque Foundation for Science, Bilbao 48009, Spain

*Corresponding authors. Email: hyosungan@jnu.ac.kr (H. An), llizmarzan@cicbiomagune.es (L. L-M.) and chris@gnu.ac.kr (J. Kim)

Materials and Methods

Figures S1–S21

Table S1–S3

Movies S1–S3

References

Materials and Methods

Section 1. Chemicals

Sodium borohydride (99%, $NaBH_4$, Sigma-Aldrich), L-ascorbic acid (BioXtra, 99%, $C_6H_8O_6$, Sigma-Aldrich), gold(III) chloride trihydrate (99.9%, $HAuCl_4·3H_2O$, Sigma-Aldrich), silver nitrate (99.0%, $AgNO_3$, Sigma-Aldrich), cetyltrimethylammonium bromide (99.9%, $C_{19}H_{42}BrN$, Sigma-Aldrich), cetylpyridinium chloride monohydrate (98%, $C_{21}H_{38}ClN·H_2O$, TCI), potassium bromide (99%, KBr, Sigma-Aldrich), hydrochloric acid (99.999%, HCl, Alfa Aesar), N,N-dimethylformamide (99.8%, C_3H_7NO, Samchun), toluene (99.7%, $C_6H_5CH_3$, Daejung), o-xylene (98%, $C_6H_4(CH_3)_2$, Samchun) and ω-thiol-terminated poly(styrene) (M_n = 11500 g/mol, 25000 g/mol, and 50000 g/mol, Polymer Source) were purchased and used without further purification. All glassware for nanoparticle (NP) syntheses was treated with aqua regia (a mixture of HCl and HNO_3), rinsed with deionized water and dried immediately before use. Deionized water (18.2 MΩ·cm at 25 °C) purified by a Merck Millipore Direct Q3 UV Water Purification System was used for all solution preparation and washing.

Section 2. Synthesis of gold NPs

2.1 Gold nanorods (NRs)

Gold NRs were prepared according to our previously reported method.[1] To synthesize gold seeds, an aqueous solution of $HAuCl_4·3H_2O$ (10 mM, 125 μL) was added to 5 mL of 100 mM cetyltrimethylammonium bromide (CTAB) and mixed well. While the mixture was stirred at 1150 rpm and an ice-cold $NaBH_4$ (10 mM, 300 μL) was quickly injected into the solution. It was stirred for 1 min, and the mixture was aged at 30 °C for 20 min. Then, aqueous solutions of $HAuCl_4·3H_2O$ (10 mM, 10 mL), $AgNO_3$ (10 mM, 1.8 mL) and ascorbic acid (100 mM, 1.14 mL) were added in sequence into 200 mL of 100 mM CTAB in a 250 mL Erlenmeyer flask while stirred at 30 °C under 200 rpm. The gold seed solution (240 μL) was added to the mixture while it was stirred at 500 rpm. The mixture was left for 2 h. After the reaction, the brown solution was centrifuged at 8000 rpm for 15 min. The supernatant was removed as much as possible, and ~36 mL of 50 mM CTAB was added to the concentrated solution combined from the six centrifuge tubes. The solution was centrifuged at 8000 rpm for 15 min and dispersed in 50 mM CTAB (λ_{max} = 682 nm).

2.2 Gold nanospheres (NSs)

Gold NSs were prepared according to our previously reported method.[2] First, the etching test was conducted to determine a volume of 10 mM $HAuCl_4$ that would be required for a spherical shape from gold NRs (Section 2.1). Different volumes of 10 mM $HAuCl_4$ solution from 3.0 to 6.0 μL were added to 0.5 mL of the gold NR (extinction = 2 at 682 nm) in 50 mM CTAB solution. They were placed in a thermomixer at 40 °C under 300 rpm for 4 h. After the reaction, an optimum volume of 10 mM $HAuCl_4$ (5.5 μL) was determined. A large-scale etching reaction was conducted as follows. The gold NR solution (4.7 mL) was mixed with 50 mM CTAB (65.39 mL) in a 250 mL Erlenmeyer flask at 40 °C, and 10 mM $HAuCl_4$ solution (771 μL) was added to the solution. It was stirred at 200 rpm for 4 h. The pink solution was centrifuged at 11000 rpm for 45 min. The supernatant was removed as much as possible, and 100 mM cetylpyridinium chloride monohydrate (CPC) was added to the concentrated solution combined from the six centrifuge tubes. The solution was centrifuged at 11000 rpm for 30 min and dispersed in 100 mM CPC (λ_{max} = 523 nm). Regrowth and re-etching processes were performed to improve the

uniformity of gold NSs. For regrowth, aqueous solutions of $HAuCl_4$ (10 mM, 583 μL), ascorbic acid (100 mM, 7.5 mL) and the as-prepared gold NS solution (11 mL, extinction = 1) was added in sequence into 33.33 mL of 10 mM CPC in a 100 mL Erlenmeyer flask while stirred at 40 °C under 300 rpm. The solution was left for 15 min, and it was centrifuged at 10000 rpm for 10 min twice and dispersed in 50 mM CTAB (λ_{max} = 534 nm). Then, re-etching was performed by reacting the regrown gold NSs (43 mL, extinction = 1) with an aqueous solution of $HAuCl_4$ (10 mM, 264 μL) at 40 °C under 200 rpm for 4 h. The solution was centrifuged at 11000 rpm for 45 min. The supernatant was removed as much as possible, and 100 mM CPC was added to the concentrated solution. It was centrifuged at 11000 rpm for 30 min and dispersed in 100 mM CPC. In order to remove relatively large-sized gold NSs, the solution was additionally centrifuged at 4000 rpm for 4 min and supernatant was collected. Centrifugation was repeated four times, and the purified gold NSs were dispersed in 100 mM CPC (λ_{max} = 523 nm).

2.3 Gold nanocubes (NCs)

Gold NCs were prepared by a seed-mediated growth method.[2] Aqueous solutions of KBr (100 mM, 0.5 mL), $HAuCl_4 \cdot 3H_2O$ (10 mM, 0.1 mL) and ascorbic acid (100 mM, 0.15 mL) were added in sequence into 5 mL of 100 mM CPC in a 20 mL Erlenmeyer flask and mixed well. Then, gold NSs prepared from Section 2.2 (30 μL, extinction = 1) were added to the mixture, and it was left for 1 h. After the reaction, the solution was centrifuged twice at 5000 rpm for 10 min and dispersed in 50 mM CTAB (λ_{max} = 611 nm).

2.4 Gold concave nanocubes (CCs)

Gold CCs were prepared by a seed-mediated growth method.[2] Aqueous solutions of HCl (1 M, 0.25 mL), $HAuCl_4 \cdot 3H_2O$ (10 mM, 0.25 mL) and $AgNO_3$ (10 mM, 62.5 μL), ascorbic acid (100 mM, 47.5 μL) were added in sequence into 5 mL of 100 mM CPC in a 20 mL Erlenmeyer flask and mixed well. Then, gold NSs prepared from Section 2.2 (50 μL, extinction = 1) were added to the mixture, and it was left for 2 h. After the reaction, the solution was centrifuged twice at 3500 rpm for 7 min and dispersed in 50 mM CTAB (λ_{max} = 668 nm).

2.5 Large-size gold NSs

Large-size gold NSs, which were used for polymer grafting, were prepared using a modified method from Section 2.2. Different volumes of 10 mM $HAuCl_4$ solution (2, 4, 6, 8, and 10 μL) were added to 0.5 mL of the gold NC solution prepared in Section 2.3 (extinction = 1 at 611 nm). Each solution was placed in a thermomixer at 40 °C under 300 rpm for 4 h. After the reaction, an optimum volume of 10 mM $HAuCl_4$ (9 μL) could be determined. Then, the gold NC solution (22 mL) was placed in a 100 mL Erlenmeyer flask at 40 °C, followed by the addition of 10 mM $HAuCl_4$ solution (396 μL). It was stirred at 200 rpm for 4 h. After the reaction, the solution was centrifuged twice at 7000 rpm for 10 min and dispersed in 50 mM CTAB (λ_{max} = 538 nm).

2.6 Au@Ag NCs

Au@Ag NCs were prepared according to a previously reported method.[3] Aqueous solutions of CPC (10 mM, 0.4 mL), ascorbic acid (50 mM, 0.2 mL) and $AgNO_3$ (20 mM, 40 μL) were added in sequence into 0.4 mL of ligand-free gold NCs in 5 mL scintillation vial at 60 °C, and it was left for 3 h. After the reaction, the solution was centrifuged twice at 5000 rpm for 10 min and dispersed in 50 mM CTAB.

Section 3. Polystyrene (PS) grafting and patch formation

3.1 PS-grafted gold NPs and PS-grafted Au@Ag NPs

PS-grafted gold NPs were prepared according to our previously reported method with modifications.[4] CTAB present in the as-prepared gold NP dispersion was gradually reduced through by two-time of centrifugation and redispersion in deionized water. After the second centrifugation, the gold NP dispersion was concentrated by removing the supernatant as much as possible (~10 μL), followed by mixing with trace amount of deionized water. At this point, the CTAB concentration was reduced to approximately 500 μM. This concentrated NP dispersion was quickly injected into the thiol-terminated PS solution (0.1 mg/mL in dimethylformamide (DMF)) while sonicating, typically for 1 min, allowing rapid surface functionalization and colloidal stabilization. The resulting sample was continuously sonicated for 3 min and left for 30 min. The PS-grafted gold NPs were collected after six-fold centrifugation and redispersed in DMF. PS-grafted Au@Ag NPs were synthesized via the aforementioned method. Detailed conditions for each core shape were shown in the table below.

Core shape	Particle volume (mL)	Polystyrene volume (mL)	Centrifugation (rpm/min)
Sphere (Au)	6.32	10	7000/10
Cube (Au)	11.94	5	5000/10
Concave cube (Au)	2.80	1	2700/7
Cube (Au@Ag)	1.30	1	7000/10

3.2 Patch formation

The solution of PS-grafted gold NPs was heated at either 90, 110, or 130 °C for 2 or 4 h. Then, the solution was slowly cooled down at room temperature for 1 h. Then, a small amount of the solution was dropped onto a substrate such as TEM grid or silicon wafer without further purification. The droplet was gently wiped after 30 min, and it was dried for 2 h before electron microscopic analysis.

Section 4. Characterization

UV-visible spectra were measured using a Genesys 10S UV-Vis spectrophotometer with a quartz cuvette (path length = 1 cm). A FEI Tecnai 12 transmission electron microscope (TEM) with a LaB_6 emitter at 120 kV, a Tescan S8000 field-emission scanning electron microscope (SEM), and a FEI Quattro S environmental-SEM (E-SEM) were used for the NP core and polymer shell characterization.

Section 5. Estimation of PS-NC concentration

The concentration of PS-NCs was estimated with that of gold cores, which could be calculated according to a previously reported literature.[5] The extinction value of 1.2 at 400 nm with gold NPs corresponds to the concentration of gold atoms as 0.5 mM. Thus, the concentration of gold atoms in our gold NCs with an extinction of 1 at 625 nm is calculated as 0.1417 mM, since their extinction value was 0.34 at 400 nm. The gold NC solution (1 mL) contains 8.53×10^{16} gold atoms/mL. In addition, the number of gold atoms per gold NC can be estimated as follows. The volume of a gold NC with 86.3 nm in edge length is calculated as 6.44×10^{5} nm^3, and the volume of a face-centered cubic unit cell consisting of 4 gold atoms is 6.79×10^{-2} nm^3. The number of gold atoms in a gold NC is calculated as 3.79×10^{7}. As we divide 3.79×10^{7}, the number of gold atoms per gold NC, by 8.53×10^{16}, the number of gold atoms per 1 mL of gold NC solution, the number of gold NC is estimated as 2.24×10^{9}. This leads

to 3.72 pM for the concentration of PS-NCs with an extinction of 1 at 625 nm. It is noted that surface ligands such as thiols may influence the extinction value at 400 nm within ~10%.[6]

Section 6. Patch angle measurement

We first drew a straight line from the center of the core NP to the farthest point on each patch, and the angle was measured as a patch angle (Figure 1c, d). One angle was measured with a particle with 2 patches, and three angles were measured with a particle with 3 patches.

Section 7. Counting of patches

The patches formed on the surface of gold NPs vary in size. For consistent patch analysis, we set up a rule as follows. Two parameters are introduced, D_{patch} (patch diameter measured at the half position between the end of the patch and the surface of the core) and L_{core} (core edge length). The patch with D_{patch} longer than half of L_{core} was only counted as a patch. The others with D_{patch} shorter than half of L_{core} were not counted as patches. Schematic illustrations are shown below.

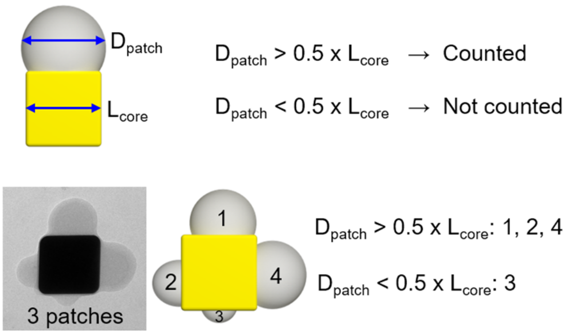

Section 8. Time-temperature superposition analysis of patch evolution

To quantitatively interpret the combined effect of time and temperature on patch evolution, a master curve was constructed by applying the time-temperature superposition principle (TTSP) based on the Arrhenius equation. The shift factor a_t was calculated using the following equation:

$$a_t = \exp\left[\frac{E_a}{R}\left(\frac{1}{T_0} - \frac{1}{T}\right)\right]$$

where E_a is the activation energy (80 kJ·mol^{-1}),[7] R is the gas constant (8.314 J·mol^{-1}·K^{-1}), T_0 is the reference temperature (90 °C, 363.15 K), and T is the experimental temperature in Kelvin. The effective time (T_{eff}) was then calculated as follows:

$$T_{\mathrm{eff}} = t \cdot a_t$$

The calculated T_{eff} values for each condition are listed below.

Condition	T_{eff} (s)	Log(T_{eff} at 90 °C)
90 °C, 2 h	7200	3.857
90 °C, 4 h	14400	4.158
110 °C, 2 h	28740	4.458

110 °C, 4 h	57481	4.760
130 °C, 2 h	99996	4.999
130 °C, 4 h	199991	5.301

Section 9. Lifted angle calculation

The projection length of the gold core in the patchy PS-NCs was measured to calculate the lifted angle of the gold core with respect to the substrate. By using two parameters, projection length (A) and core edge length (B), the lifted angle can be calculated by the following equation: $A = B(\cos\theta + \sin\theta)$. Schematic illustrations are shown below. It is noted that the core edge length value was used with 86.3 nm that was derived from the TEM images, since it was difficult to be measured in the SEM images.

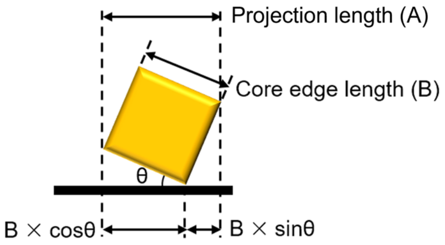

Section 10. Patch contact area and facet coverage calculation

The contact diameter of each polymer patch was measured from SEM images by drawing a straight line across the base of the patch. The contact area was calculated assuming circular geometry. Surface coverage was estimated by dividing the calculated contact area by the area of a single cube facet (7448 nm^2), which was determined using the average edge length (86.3 nm) measured from TEM images.

Section 11. Molecular dynamics simulations

All-atom molecular dynamics (MD) simulations were performed using GROMACS (version 2022.3)[8] and the CHARMM36 force field.[9] To simulate the polymer, we have chosen an atactic thiol-PS chain containing 40 monomer units. The force field parameters for PS were generated with the CGenFF program[10] and the Lennard-Jones parameters for gold were those reported by Heinz *et al.*[11] which have been proven to provide good descriptions for systems adsorbed on gold surfaces.

The brush model for PS adsorbed on a gold surface was assembled by using 20 PS chains interacting with an Au(100) slab of $5.3 \times 5.3 \times 1.1$ nm^3 containing 2028 gold atoms which resulted in a grafting density of 0.8 chains/nm^2. A model for PS adsorbed on a cubic gold NP was constructed employing 48 PS chains and a cube of 2.5 nm edge. The grafting density for this latter model (1.28 chain/nm^2) is slightly larger because the area and volume available for the PS chains grafted to the NC metal increases with the curvature of the shape NP. Only for comparison purposes, two additional models of PS grafted on octahedral spherical gold were also constructed: A small octahedral with a grafting density similar to the initial brush model and a sphere (the limit of higher curvature) interacting with the same number of PS chains than the NC. As starting points for the MD simulations, the thiol groups of the PS chains were oriented towards the metal with their sulfur atoms located at distances around 3 Å from the surface and the structures of the gold units have been fixed during the simulations by using very high

Cartesian restraints (10000 kJ·mol^{-1}·nm^{-2}).

After assembling each system, an initial energy minimization was performed until the maximum force on any atom is lower than 1000 kJ·mol^{-1}·nm^{-1}, followed by NVT and NPT equilibrations during 1 ns with a time step of 1 fs. The temperature was initially kept constant at 300 K employing the V-rescale thermostat, the pressure was kept at 1 atm using the Parrinello-Rahman barostat and the cutoff for van der Waals interactions was set at 1.2 nm. The assembled systems were equilibrated by means of an initial NPT production run at 300 K for 10 ns. To populate the conformational space of the PS polymer, a slight heating at 450 K and cooling down at the initial 300 K (5 ns) was performed and followed by an additional NPT equilibration run of 25 ns. To mimic the experimental procedure, the equilibrated systems were heated at different temperatures until the detaching of the PS chains was observed. The volume of the system during the heating step was kept constant by adjusting the height of the simulation box (brush model) and the size of the simulation box to avoid interactions between adjacent cells. Finally, the detached configurations were cooled down until 300 K and a final NVT equilibration run was performed during at least 25 ns. The trajectories for the last 5 ns of the equilibrated systems before and after heating were used for data analysis. It must be noted here that the large size of the simulation boxes employed for the heating steps prevented the inclusion of solvent in the simulations.

Figures S1–S21

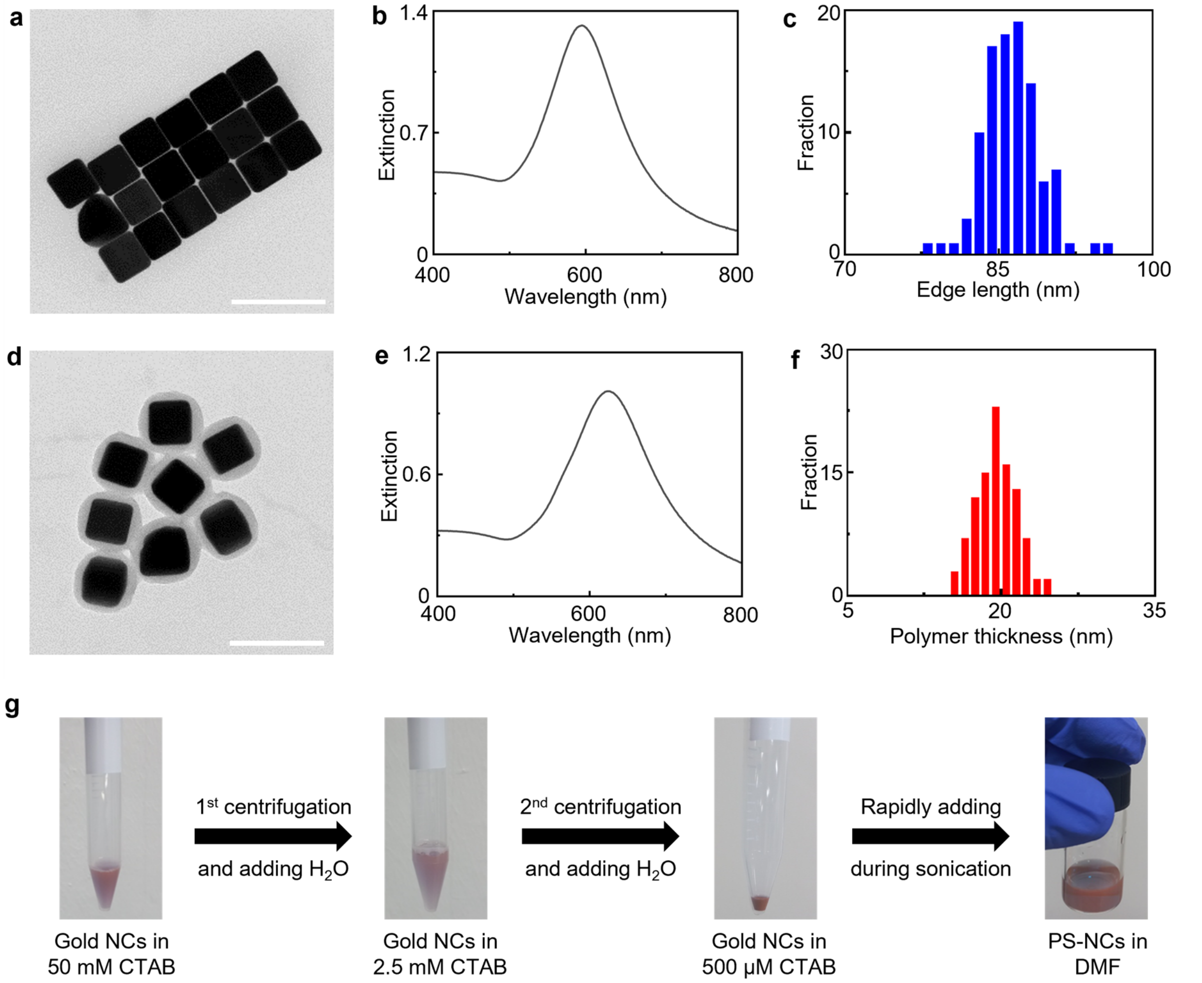

Figure S1. (a) A representative TEM image, (b) UV-visible spectrum, and (c) edge length distribution histogram of gold NCs. (d) A representative TEM image, (e) UV-visible spectrum, and (f) polymer shell thickness distribution histogram of PS-NCs. (g) Photograph of sequential washing steps for removing CTAB prior to polymer grafting on gold NCs. Scale bars: 200 nm.

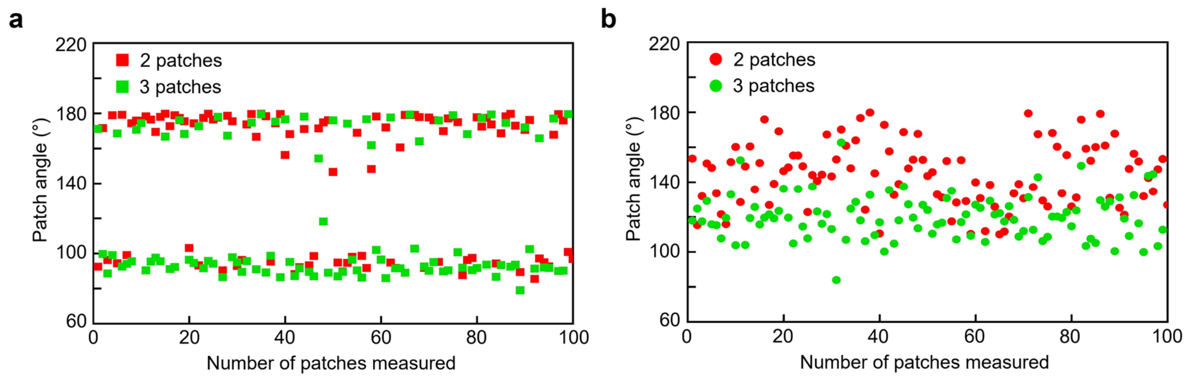

Figure S2. (a) Plot of patch angle values measured from patchy PS-NCs with two (red) and three (green) patches. (b) Plot of patch angle values measured from patchy PS-NSs with two (red) and three (green) patches.

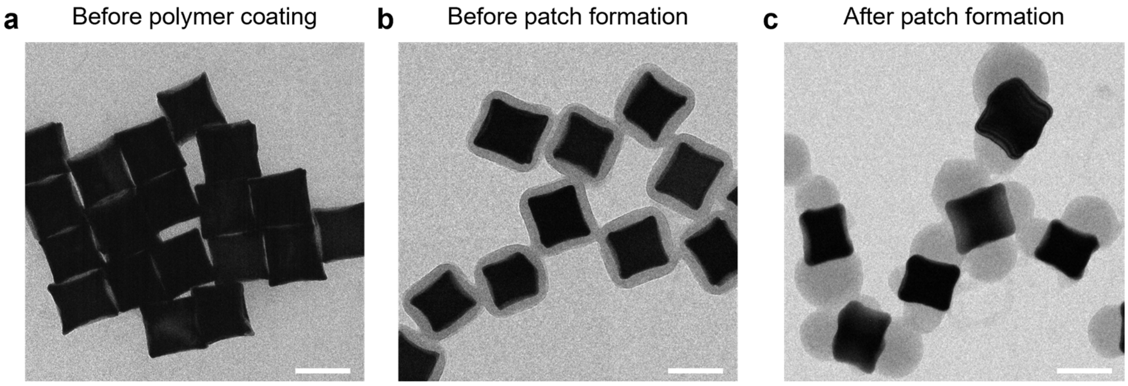

Figure S3. Representative TEM images of (a) gold CCs, (b) PS-CCs before and (c) after heating at 90 °C for 2 hours. Scale bars: 100 nm.

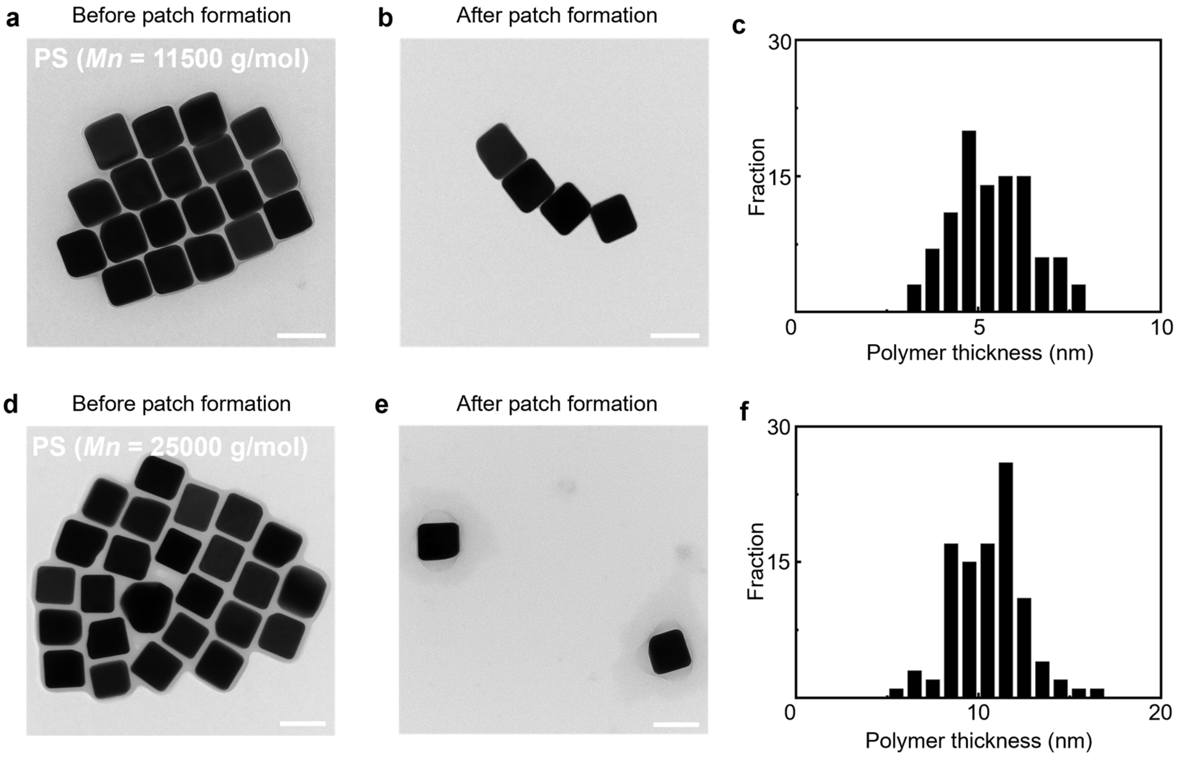

Figure S4. Representative TEM images of gold NCs grafted with PS (*Mn* = 11500 g/mol) (a) before patch formation and (b) after patch formation, and (c) histogram of PS shell thickness before patch formation. Representative TEM images of gold NCs grafted with PS (*Mn* = 25000 g/mol) (d) before patch formation and (e) after patch formation, and (f) histogram of PS shell thickness before patch formation. Scale bars: 100 nm.

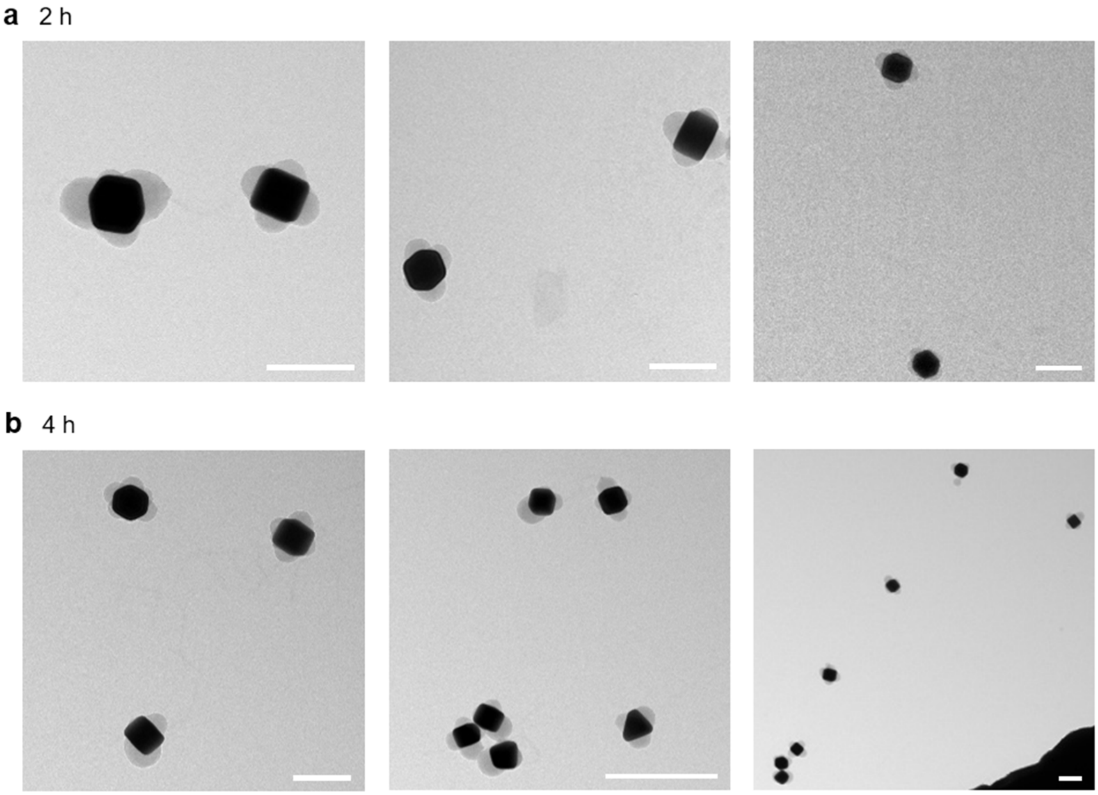

Figure S5. Representative TEM images of the patchy PS-NCs after heating at 90 °C for 2 hours (a) and 4 hours (b). Scale bars: 200 nm.

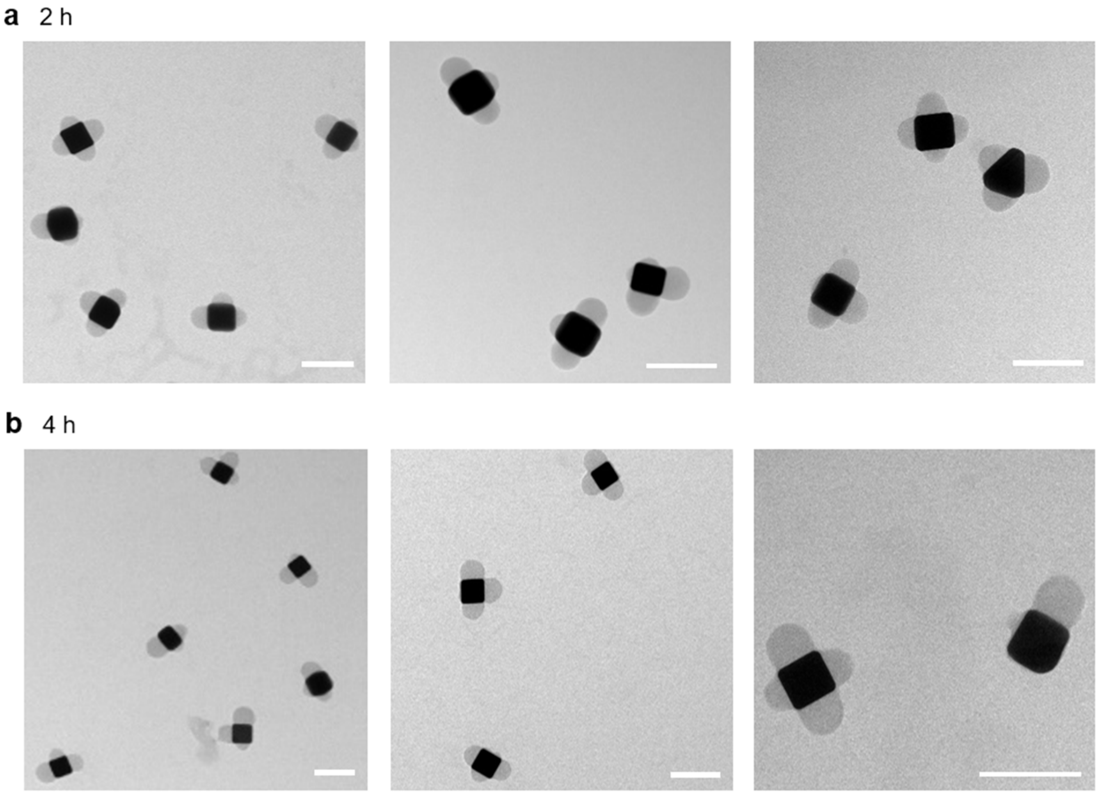

Figure S6. Representative TEM images of the patchy PS-NCs after heating at 110 °C for 2 hours (a) and 4 hours (b). Scale bars: 200 nm.

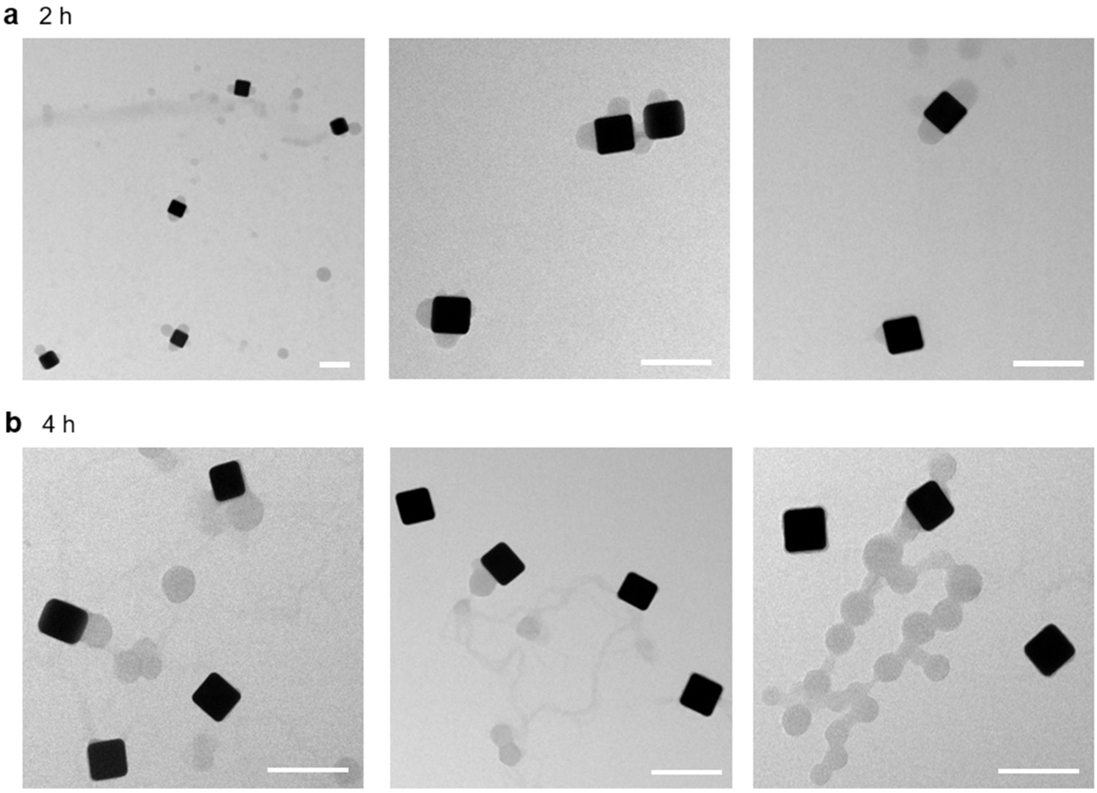

Figure S7. Representative TEM images of the patchy PS-NCs after heating at 130 °C for 2 hours (a) and 4 hours (b). Scale bars: 200 nm.

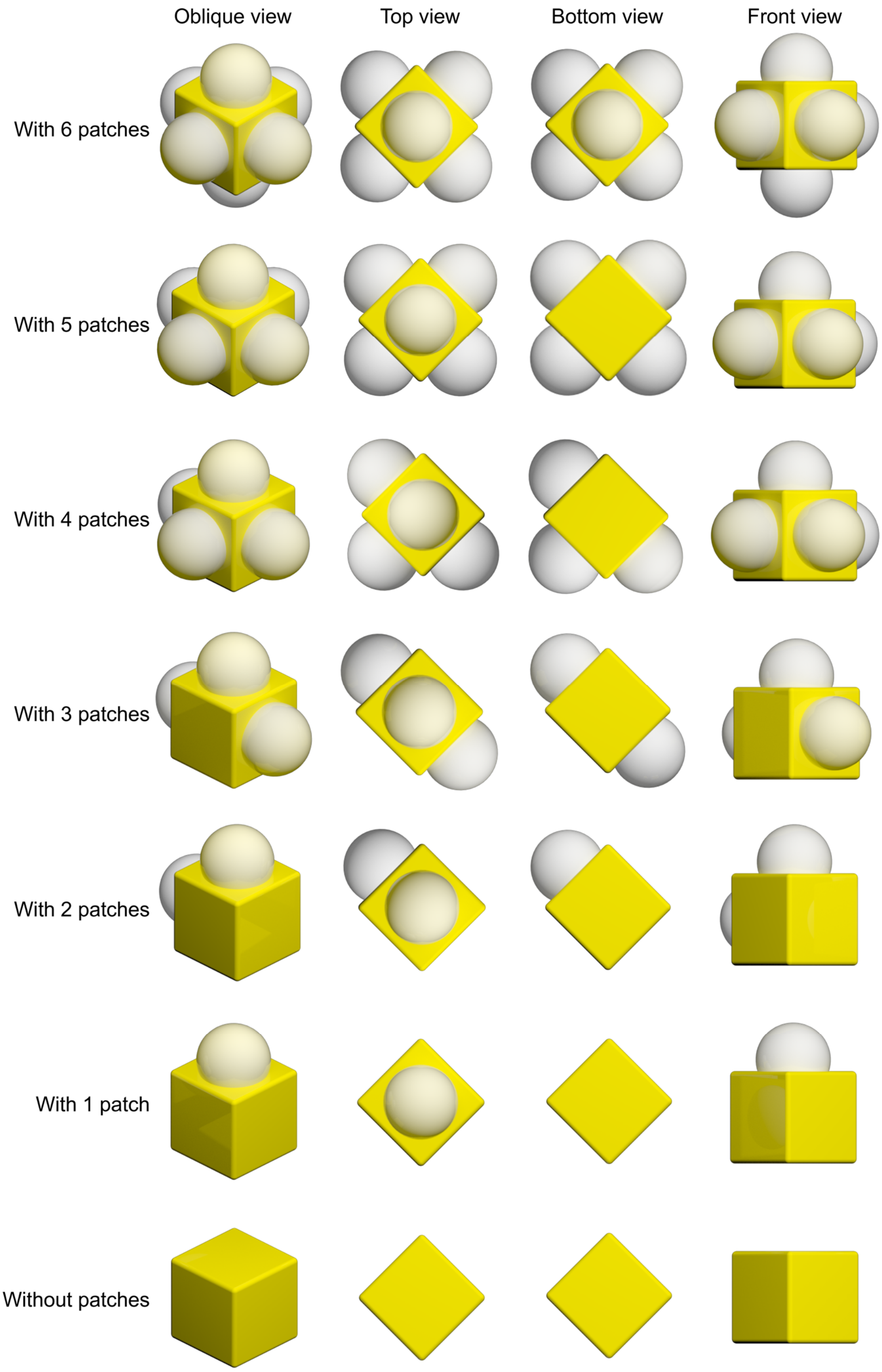

Figure S8. Representative 3D models of patchy PS-NCs with 0 to 6 patches, visualized from oblique, top, bottom, and front views. These orientations demonstrate that patches located directly above or below the NC core may be hidden in 2D TEM images, potentially leading to slight under-counting. Since the NCs are randomly oriented on the grid, this effect is expected to be non-systematic and does not significantly alter the observed distribution trends.

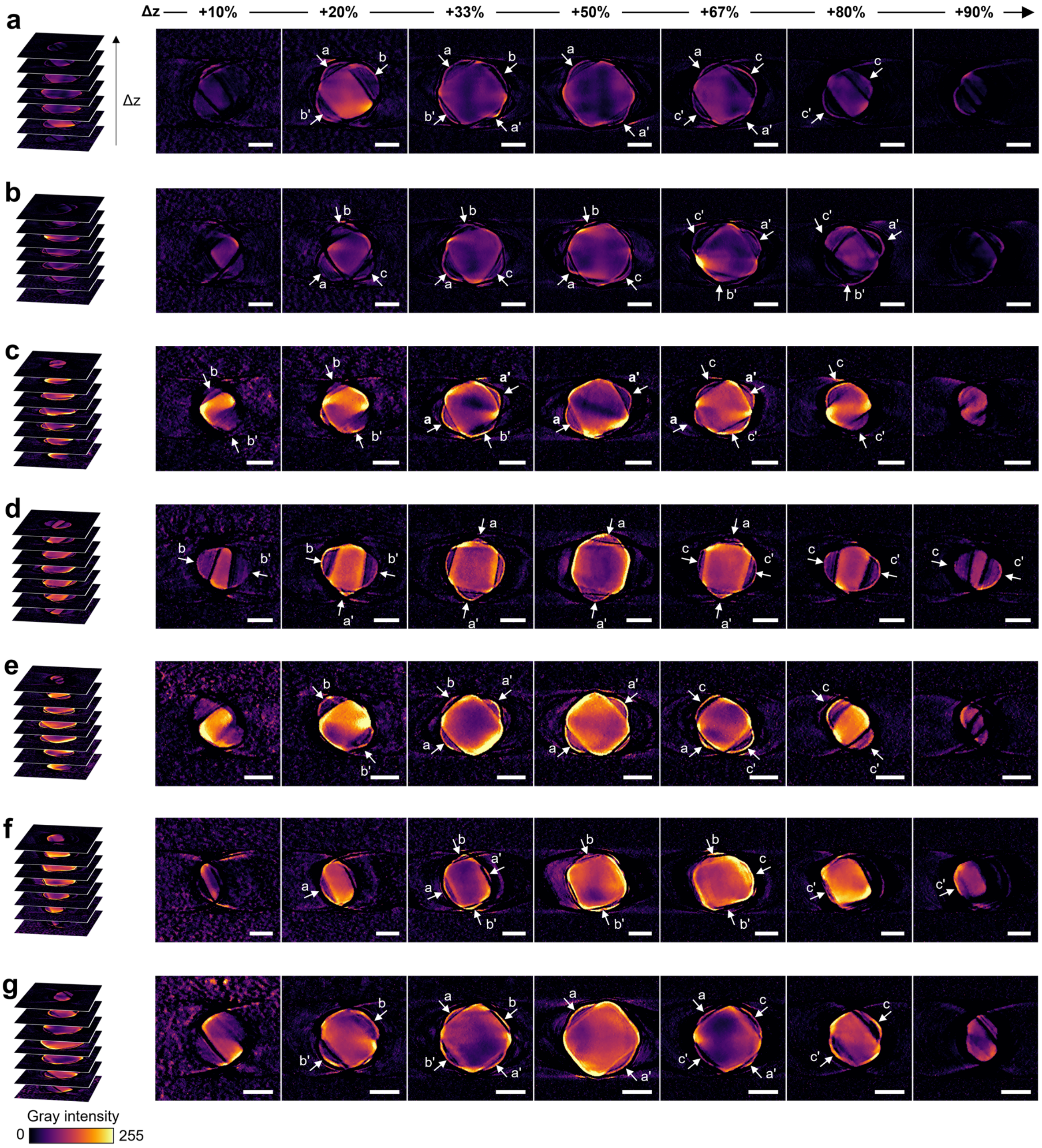

Figure S9. The z-slices for the reconstructed tomograms for seven different patchy PS-NCs after heating at 90 °C for 2 hours. Each NC contains six patches, which are indicated by the arrows. Scale bars: 50 nm.

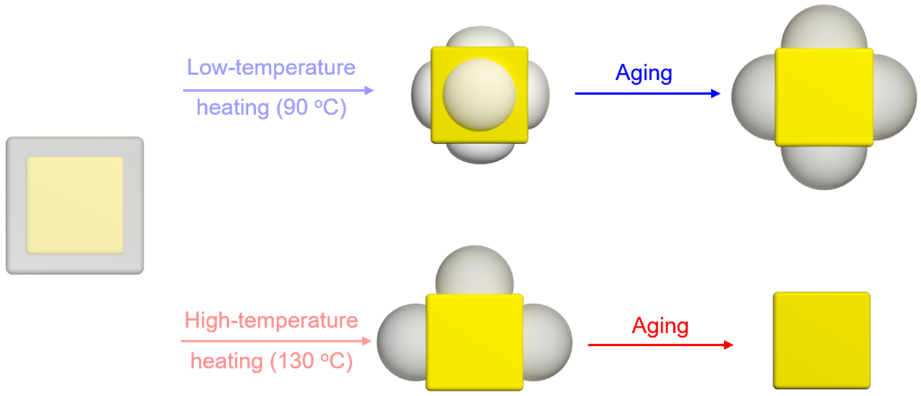

Figure S10. Schematic illustration for the thermally-driven patch formation process. At moderate temperature (90 °C), desorbed PS chains migrate and reassemble into patches on low-curvature surfaces during aging. At elevated temperature (130 °C), although patches initially form, prolonged heating results in further Au-S bond dissociation even at low-curvature regions, leading to detachment of entire polymer patches from the nanoparticle surface.

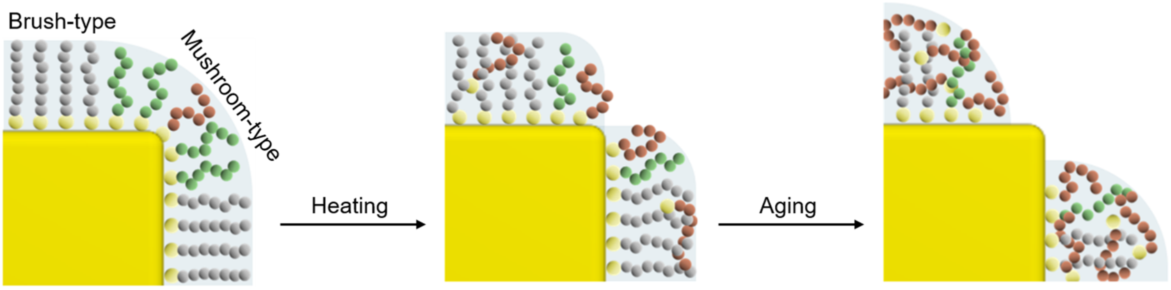

Figure S11. Schematic illustration for the preferential desorption of PS layers from high-curvature surfaces and their reattachment onto the existing brush-type PS layers on low-curvature surfaces.

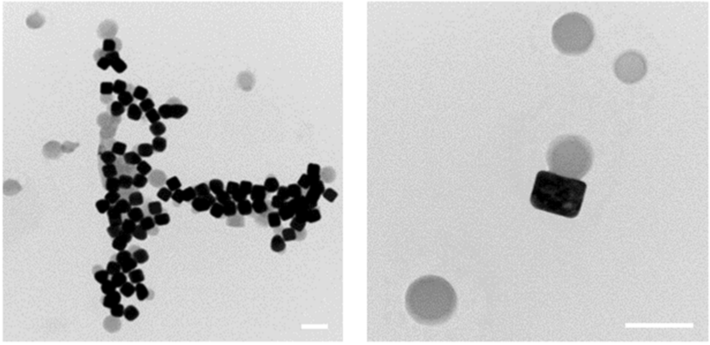

Figure S12. Representative TEM images of the patchy PS-NCs after heating at 90 °C for 2 hours under mechanical stirring at 100 rpm. Scale bars: 200 nm.

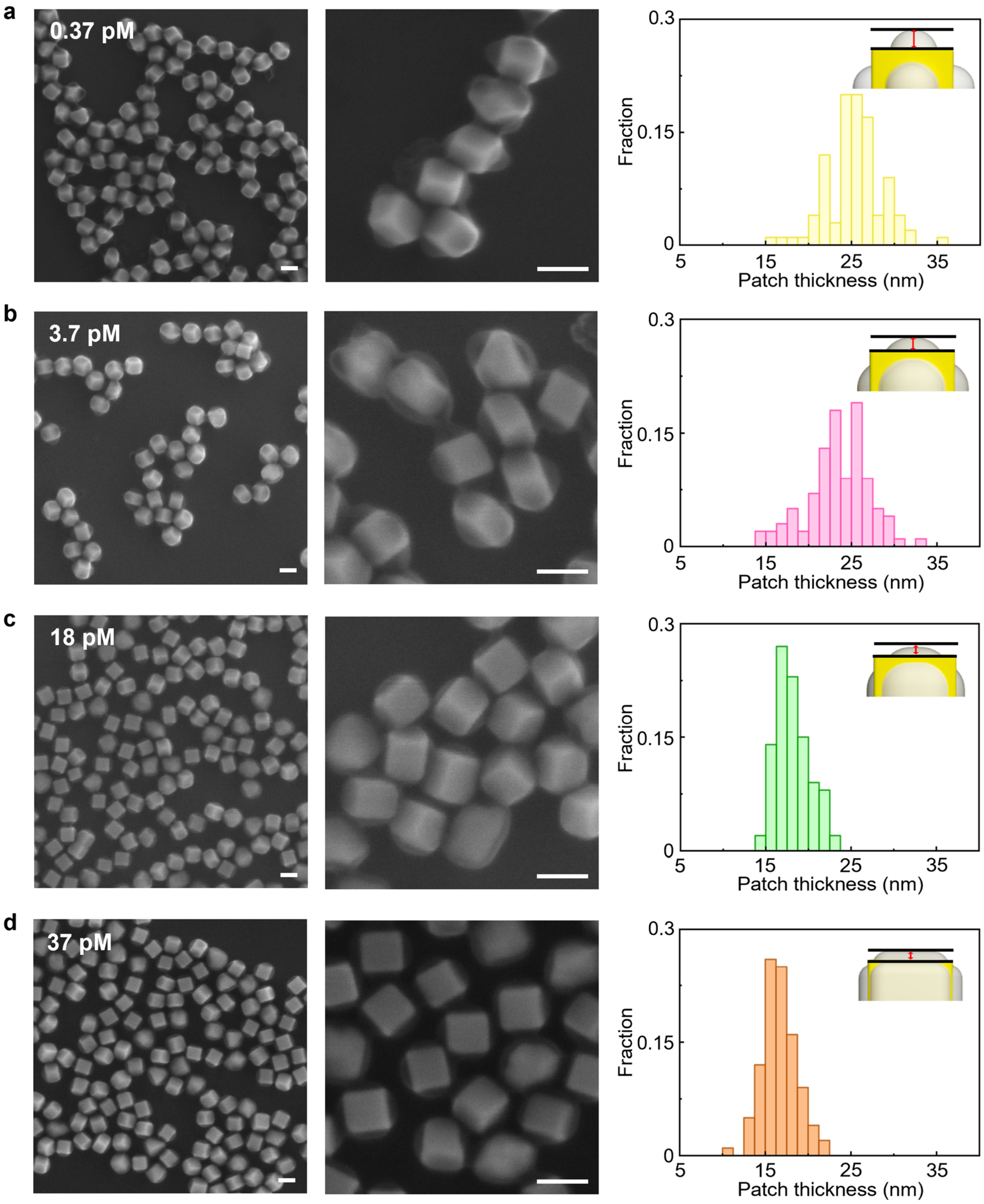

Figure S13. Representative SEM images and patch thickness distribution histograms of the patchy PS-NCs after heating at 90 °C for 2 hours in concentration of (a) 0.37 pM, (b) 3.7 pM, (c) 18 pM, and (d) 37 pM. Scale bars: 100 nm.

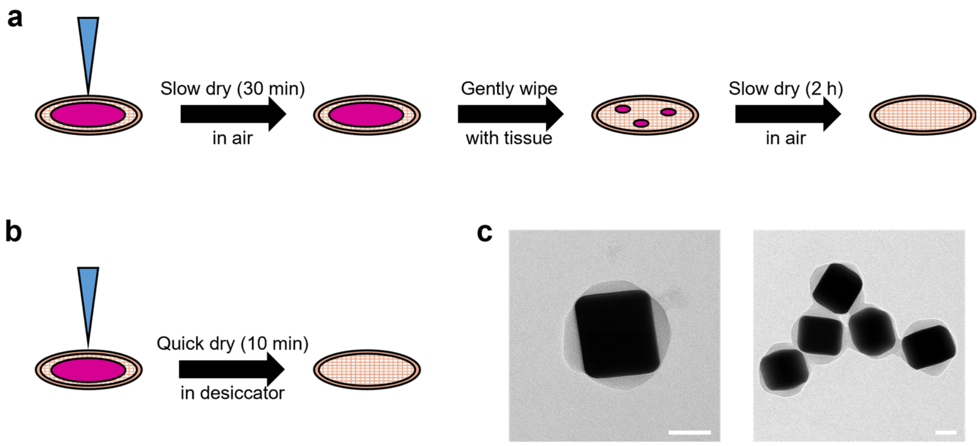

Figure S14. (a) Illustration of the slow drying process in air, including a drying step of 30 min, gentle wiping with tissue, followed by 2 hours of drying. (b) Illustration of the quick drying process in a desiccator with a vacuum pump for 10 min. (c) TEM images showing patchy PS-NCs after heating at 90 °C for 2 hours, prepared by the drying method described in (b). Scale bar: 50 nm.

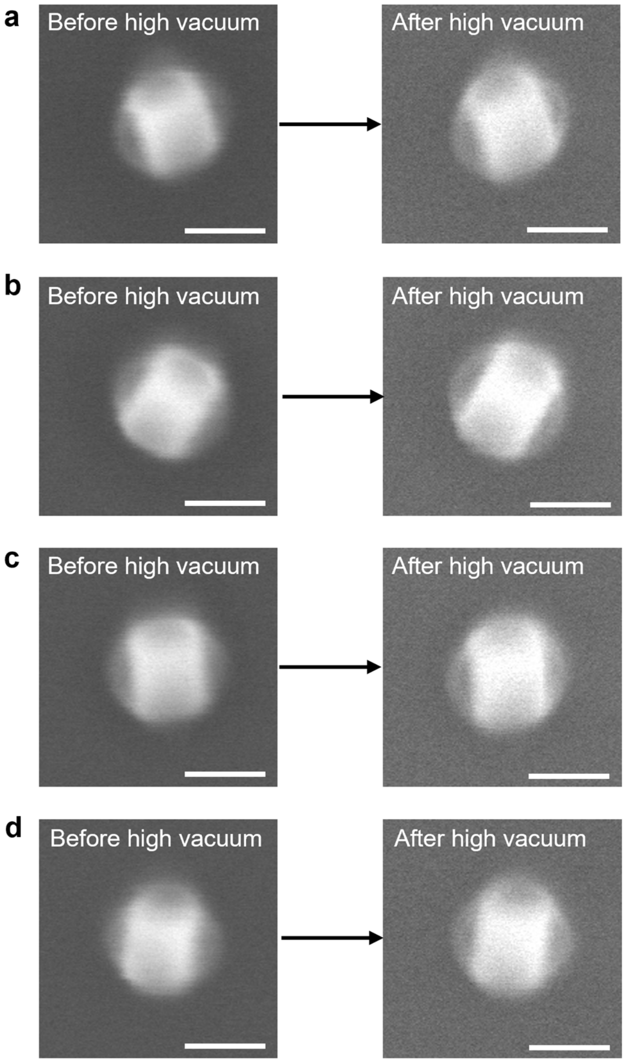

Figure S15. (a–d) Representative E-SEM images of the patchy PS-NCs after heating at 90 °C for 2 hours. The patchy PS-NCs were initially imaged under low-vacuum mode (10^3 Pa), and subsequently high-vacuum mode (10^{-3} Pa) was applied to the same specimen for ~30 min. All the images before and after high vacuum were obtained under low-vacuum mode. Scale bars: 100 nm.

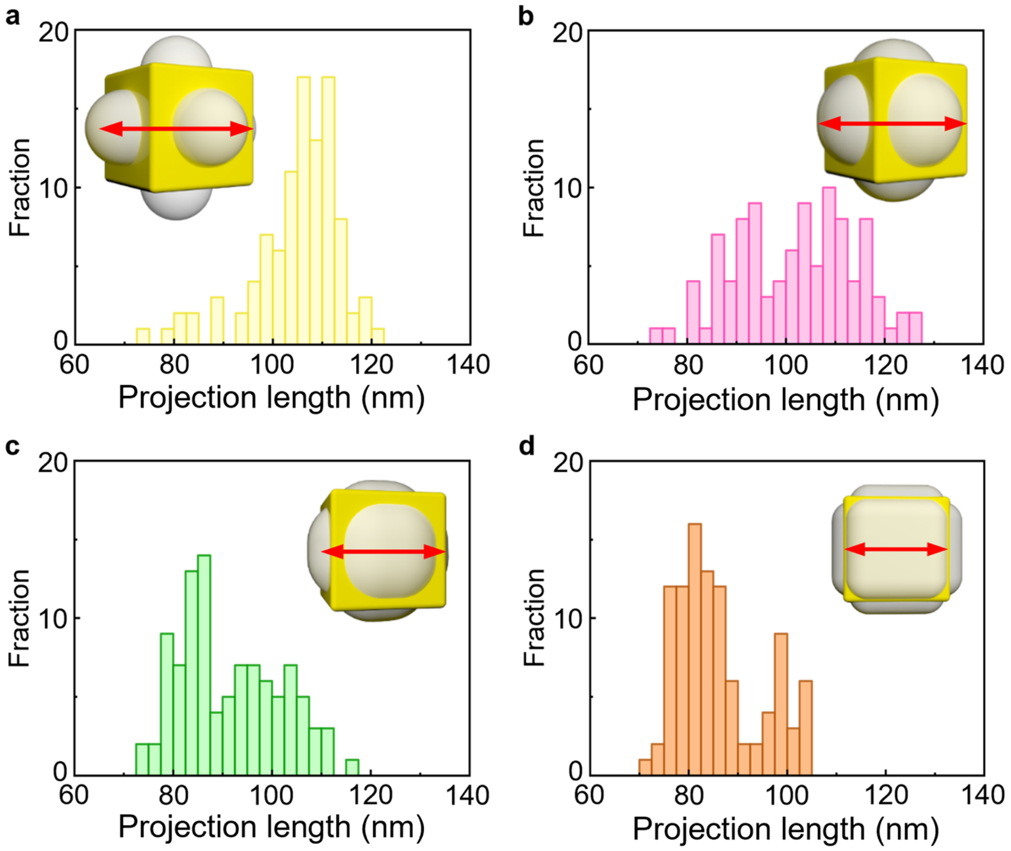

Figure S16. Projection length distribution histograms of the patchy PS-NCs after heating at 90 ℃ for 2 hours in concentration of (a) 0.37 pM, (b) 3.7 pM, (c) 18 pM, and (d) 37 pM.

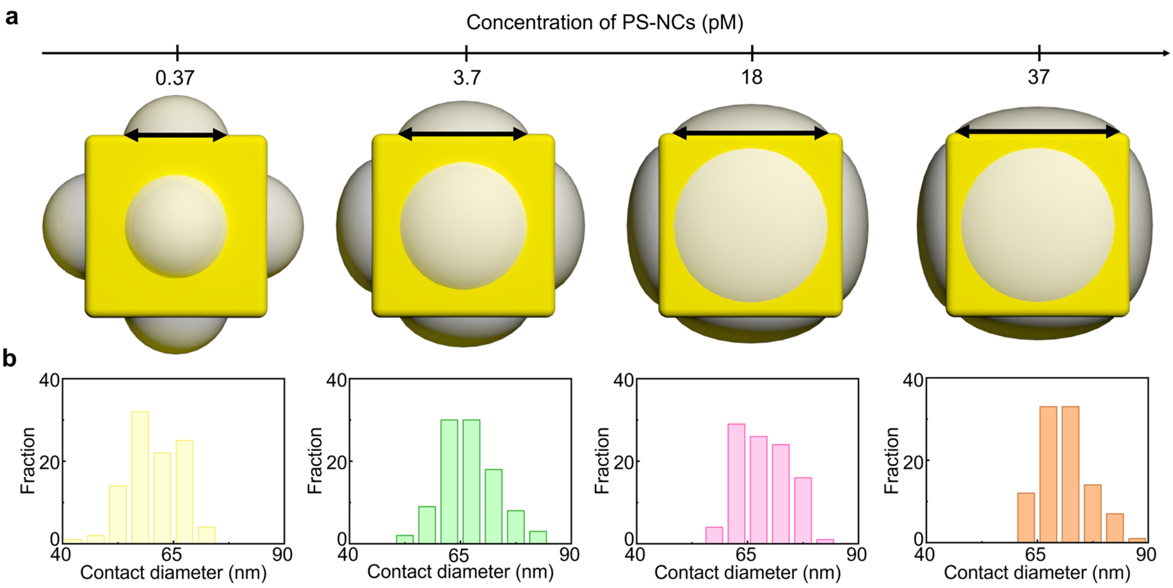

Figure S17. (a) Schematic illustrations of patchy PS-NCs after thermal treatment at different particle concentrations (0.37, 3.7, 18, and 37 pM from left to right), showing an increase in patch contact diameter with increasing concentration. (b) Corresponding histograms of measured contact diameters from SEM analysis at each concentration. The observed trend supports the concentration-dependent redistribution of PS chains, where lower concentrations promote the formation of thicker and more convex patches on low-curvature surfaces.

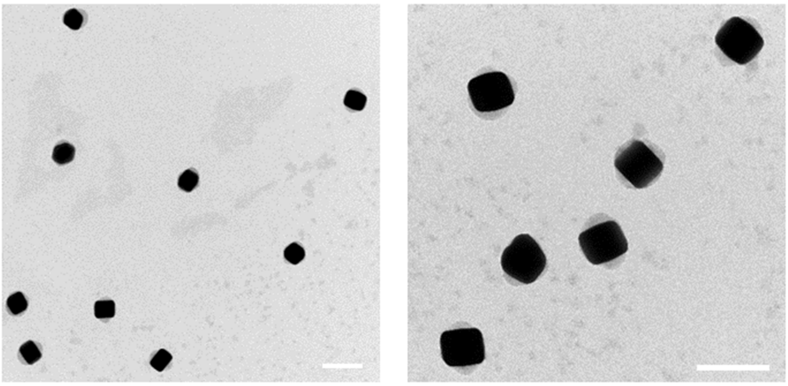

Figure S18. Representative TEM images of the patchy PS-NCs after heating at 90 ℃ for 2 hours in a mixture solvent of DMF and toluene. Scale bars: 200 nm.

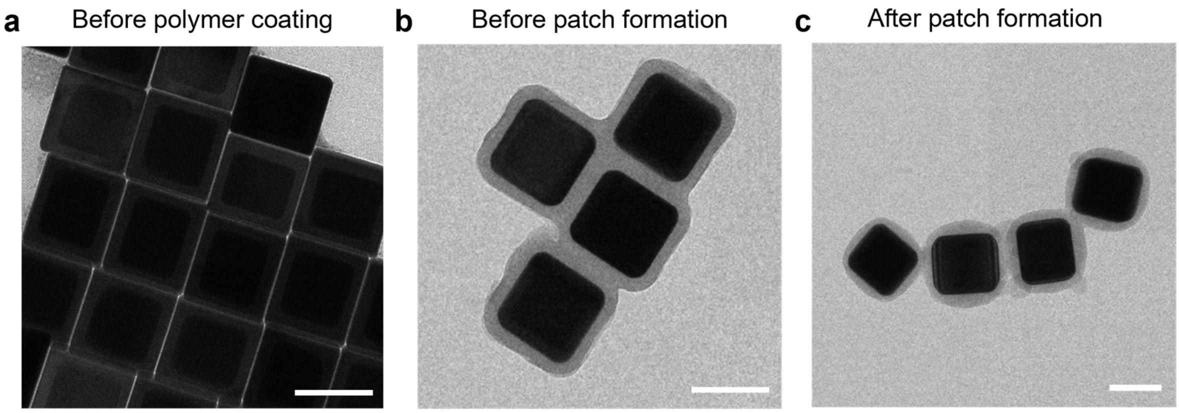

Figure S19. Representative TEM images of (a) Au@Ag NCs, (b) PS-Au@Ag NCs, and (c) PS-Au@Ag NCs after heating at 90 °C for 2 hours. Scale bars: 100 nm.

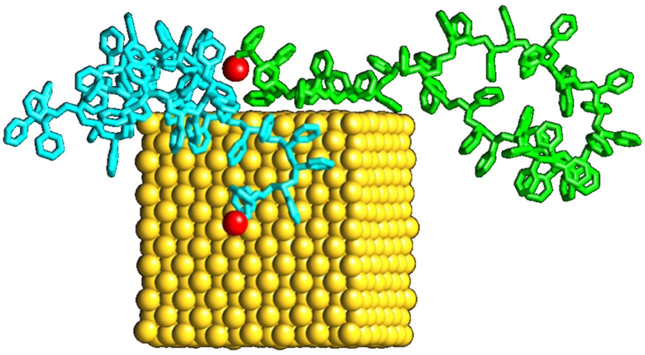

Figure S20. Detailed depiction for two highlighted PS chains adsorbed on a gold NC showing the interactions between the lateral chains of the polymers and the metallic surface.

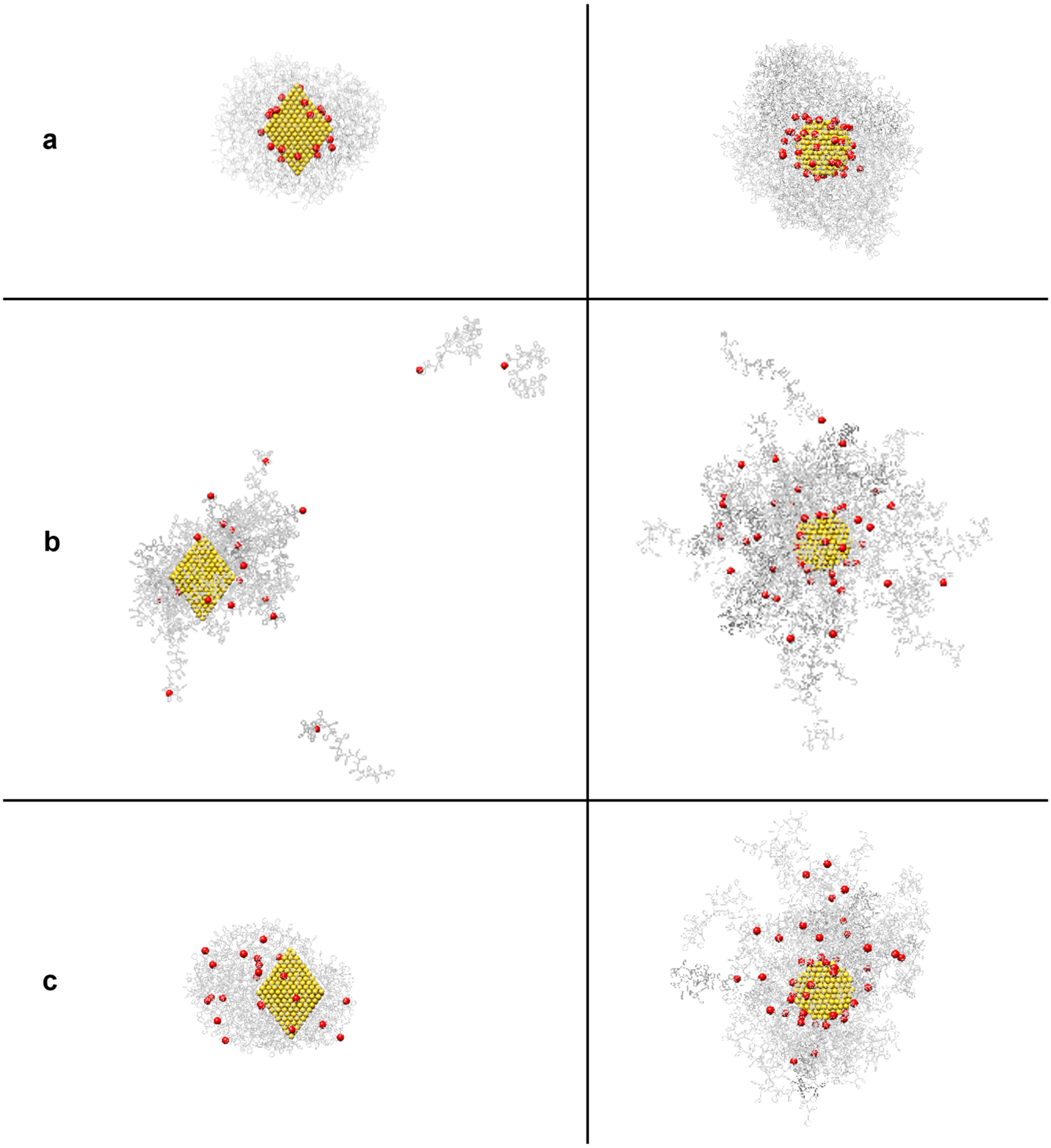

Figure S21. Selected snapshot for MD simulations of PS chains adsorbed on gold NPs of different shape (left column: octahedron; right column: sphere). (a) Initial symmetric disposition at room temperature. (b) Intermediate arrangement at the end of the heating step showing the detaching of the polymer. (c) Final asymmetric configuration around the metal NP after cooling down to the initial temperature. PS chains are represented in gray color. Sulfur atoms are depicted as red spheres.

Table S1. Contact diameter, contact area, and surface coverage of polymer patches measured from patchy PS-NCs at different concentrations shown in Figure 4a.

Concentration (pM)	Contact diameter (nm)	Contact area (nm^2)	Coverage (%)
0.37	60.6	2880	38.7
3.7	67.0	3524	47.3
18	68.6	3691	49.6
37	71.1	3968	53.3

Table S2. (a) Details of the models for PS chains interacting with different gold systems. (b) Initial configuration for MD simulations of each model.

a

Number of PS chains	Gold model	Number of gold atoms	Total number of atoms	Grafting density (chains/nm^2)
20	Slab<br>5.1 × 5.1 × 1.1 nm^3<br>Box height: 50 nm	2028	14888	0.77
48	Cube<br>2.5 nm edge<br>Box edge: 230 nm	1099	31963	1.28
24	Octahedron<br>2.7 nm edge<br>Box edge: 250 nm	891	16323	0.82
48	Sphere<br>1.25 nm radius<br>Box edge: 250 nm	555	31419	2.44

b

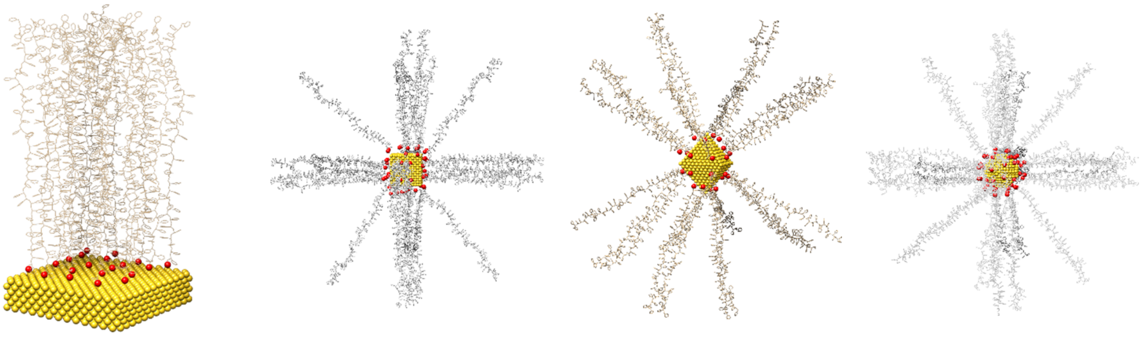

Table S3. PS end-to-end distance (R_{ee}), radius of gyration (R_g) and the components along the three principal axes for (a) the 20PS and 48PS models of free polymers, (b) the model of 20 PS adsorbed on a flat gold surface before and after heating and (c) the model of 48 PS adsorbed on the gold NC before and after heating.

		R_{ee} (nm)	R_g(nm)	R_g^1	R_g^2	R_g^3
**a**	20PS	3.149	1.321	0.875	0.731	0.668
	48PS	3.579	1.412	0.904	0.803	0.729
**b**	Au(flat)@20PS					
	Before heating	4.730	1.663	1.442	0.603	0.567
	After heating	4.030	1.525	1.130	0.795	0.644
**c**	Au(cube)@48PS					
	Before heating	2.688	1.207	0.761	0.690	0.633
	After heating	3.085	1.363	0.859	0.794	0.698

Movies S1–S3

Movie S1. A series of TEM tilt images of two patchy PS-NCs with 6 patches.

Movie S2. 3D reconstruction and schematic model of a patchy PS-NC with 6 patches.

Movie S3. Z-slice series of two patchy PS-NCs with 6 patches.

References

[1] J. Lee, H. Lee, C. Kiguye, C. Bae, J. Kim, Controllable Coating and Reshaping of Gold Nanorods with Tetracyanoquinodimethane. *Chem. Commun.* **2019**, *55*, 11731.

[2] M. N. O'Brien, M. R. Jones, K. A. Brown, C. A. Mirkin, Universal Noble Metal Nanoparticle Seeds Realized Through Iterative Reductive Growth and Oxidative Dissolution Reactions. *J. Am. Chem. Soc.* **2014**, *136*, 7603.

[3] A. Klinkova, H. Thérien-Aubin, A. Ahmed, D. Nykypanchuk, R. M. Choueiri, B. Gagnon, A. Muntyanu, O. Gang, G. C. Walker, E. Kumacheva, Structural and Optical Properties of Self-Assembled Chains of Plasmonic Nanocubes. *Nano Lett.* **2014**, *14*, 6314.

[4] J. Lee, C. Bae, Z. Ou, S. Park, J. Kim, J. Kim, Nanoscopic Morphological Effect on the Optical Properties of Polymer-Grafted Gold Polyhedra. *Nanoscale Adv.* **2021**, *3*, 1927.

[5] L. Scarabelli, A. Sánchez-Iglesias, J. Pérez-Juste, L. M. Liz-Marzán, A "Tips and Tricks" Practical Guide to the Synthesis of Gold Nanorods. *J. Phys. Chem. Lett.* **2015**, *6*, 4270.

[6] T. Hendel, M. Wuithschick, F. Kettemann, A. Birnbaum, K. Rademann, J. Polte, In Situ Determination of Colloidal Gold Concentrations with UV–Vis Spectroscopy: Limitations and Perspectives. *Anal. Chem.* **2014**, *86*, 11115.

[7] F. Li, H. Zhang, B. Dever, X.-F. Li, X. C. Le, Thermal Stability of DNA Functionalized Gold Nanoparticles. *Bioconjugate Chem.* **2013**, *24*, 1790

[8] M. J. Abraham, T. Murtola, R. Schulz, S. Páll, J. C. Smith, B. Hess, E. Lindahl, GROMACS: High Performance Molecular Simulations through Multi-Level Parallelism from Laptops to Supercomputers. *SoftwareX* **2015**, *1*, 19.

[9] K. Vanommeslaeghe, E. Hatcher, C. Acharya, S. Kundu, S. Zhong, J. Shim, E. Darian, O. Guvench, P. Lopes, I. Vorobyov, A. D. Mackerell Jr., CHARMM General Force Field: A Force Field for Drug-like Molecules Compatible with the CHARMM All-Atom Additive Biological Force Fields. *J. Comput. Chem.* **2010**, *31*, 671.

[10] K. Vanommeslaeghe, A. D. MacKerell Jr., Automation of the CHARMM General Force Field (CGenFF) I: Bond Perception and Atom Typing. *J. Chem. Inf. Model.* **2012**, *52*, 3144.

[11] H. Heinz, R. A. Vaia, B. L. Farmer, R. R. Naik, Accurate Simulation of Surfaces and Interfaces of Face-Centered Cubic Metals Using 12−6 and 9−6 Lennard-Jones Potentials. *J. Phys. Chem. C* **2008**, *112*, 17281.
